# Muscle regulates mTOR dependent axonal local translation in motor neurons via CTRP3 secretion: implications for a neuromuscular disorder, spinal muscular atrophy

**DOI:** 10.1186/s40478-019-0806-3

**Published:** 2019-10-15

**Authors:** Wiebke A. Rehorst, Maximilian P. Thelen, Hendrik Nolte, Clara Türk, Sebahattin Cirak, Jonathan M. Peterson, G. William Wong, Brunhilde Wirth, Marcus Krüger, Dominic Winter, Min Jeong Kye

**Affiliations:** 1 Institute of Human Genetics, University of Cologne, Kerpener Str. 34, 50931 Cologne, Germany; 2 Excellence Cluster on Cellular Stress Responses in Aging Associated Diseases (CECAD), University of Cologne, Cologne, Germany; 3 Department of Pediatrics, University Hospital Cologne, Cologne, Germany; 4 Center for Molecular Medicine Cologne, University of Cologne, Cologne, Germany; 5 Department of Health Sciences, College of Public Health and the Department of Biomedical Sciences, Quillen College of Medicine, East Tennessee State University, Johnson City, TN USA; 6 Department of Physiology, The Johns Hopkins University School of Medicine, Baltimore, MD USA; 7 Institute for Genetics, University of Cologne, Cologne, Germany; 8 Center for Rare Disease Cologne, University Hospital Cologne, Cologne, Germany; 9 Institute of Biochemistry and Molecular Biology, University of Bonn, Bonn, Germany

**Keywords:** Spinal muscular atrophy, SMN (survival motor neuron), Muscle secretome, Neuronal protein synthesis, CTRP3, Motor neuron disease

## Abstract

Spinal muscular atrophy (SMA) is an inherited neuromuscular disorder, which causes dysfunction/loss of lower motor neurons and muscle weakness as well as atrophy. While SMA is primarily considered as a motor neuron disease, recent data suggests that survival motor neuron (SMN) deficiency in muscle causes intrinsic defects. We systematically profiled secreted proteins from control and SMN deficient muscle cells with two combined metabolic labeling methods and mass spectrometry. From the screening, we found lower levels of C1q/TNF-related protein 3 (CTRP3) in the SMA muscle secretome and confirmed that CTRP3 levels are indeed reduced in muscle tissues and serum of an SMA mouse model. We identified that CTRP3 regulates neuronal protein synthesis including SMN via mTOR pathway. Furthermore, CTRP3 enhances axonal outgrowth and protein synthesis rate, which are well-known impaired processes in SMA motor neurons. Our data revealed a new molecular mechanism by which muscles regulate the physiology of motor neurons via secreted molecules. Dysregulation of this mechanism contributes to the pathophysiology of SMA.

## Introduction

Spinal muscular atrophy (SMA) is an inherited neuromuscular disorder with an incidence of 1 per 6000–10,000 newborns [66, 72]. While the severity of SMA varies, characteristic phenotypes of SMA patients include muscle weakness and atrophy, defects in neuromuscular junctions (NMJs) and motor neuron loss. In more than 95% of cases, SMA is caused by loss or mutations of the *SMN1* (Survival of Motor Neuron 1) gene. In addition to *SMN1*, the human genome contains a copy gene *SMN2*, which produces approximately 10% of SMN protein compared to *SMN1* due to a point mutation in exon7 [40, 45]. As the severity of SMA is highly correlated with SMN protein levels, the presence of additional *SMN2* genes determines the severity of the disease [22, 41]. Rarely, SMN independent genetic modifiers are reported as influencing factors for the severity of SMA [18, 29, 55, 60]. Primarily, SMA is considered as a motor neuron disease with severe defects in NMJs and motor neurons, leading to muscle wasting. However, growing amount of evidence indicates that the severe type I SMA cases, with one or two copies of *SMN2,* show multi-organ defects [25]. Only in the milder cases of SMA, with 3–6 copies of *SMN2*, are pathological symptoms restricted to motor neurons, NMJs and muscles [75]. Nevertheless, it is not clear whether moderate defects in other organs contribute to the fitness of NMJs and motor neurons even in the mild forms of SMA.

The NMJ is the synapse between motor neuron axons and muscle fibers. As it plays a critical role in muscle function, even minor dysfunction of the NMJ can cause devastating outcomes such as muscle weakness and atrophy [68]. At mature NMJs presynaptic motor neuron axon terminals release acetylcholine to induce muscle contraction by binding to acetylcholine receptors, which form clusters at postsynaptic muscle fibers to potentiate the responsiveness. While most NMJs stay remarkably stable during adulthood in animals, the mature NMJs also require dynamic denervation and re-innervation processes to maintain their structure and functionality. For example, it is known that muscle fibers release chemotactic signals for motor axons to re-innervate during this process [24]. Dysfunction and degeneration of NMJs due to genetic or other pathological reasons cause neuromuscular diseases. At the presynaptic site, it may cause motor neuron loss via dying-back mechanisms, and at the post-synaptic muscle site, it causes muscle weakness and atrophy. In case of a severe SMA mouse model, NMJ dysfunction was observed in 4 days after birth, whereas motor neuron loss was found in 14 days old mice [35]. While it is well characterized how motor neurons regulate muscle function, it is less understood how muscles contribute to neuronal physiology, especially in motor neuron diseases.

SMA motor neurons exhibit characteristic phenotypes. In vitro, these phenotypes include impaired growth [61], dysregulated splicing and miRNA processing [15, 23], reduced protein synthesis efficiency [1, 21, 37], impaired energy metabolism [7, 47], enhanced excitability and impaired Ca^2+^ homeostasis together with mis-localized Ca^2+^ channels [31]. Furthermore, well-described functions of SMN are the biogenesis of small nuclear ribonucleoproteins (snRNPs) [79] and trafficking of mRNAs to axon terminals [1, 21]. Among these phenotypes of SMA motor neurons, reduced protein synthesis efficiency may contribute to impaired growth. Importantly, dysregulated protein synthesis has been also reported in other neurological disorders such as Alzheimer’s disease, Charcot-Marie-Tooth and Amyotrophic lateral sclerosis [3, 34, 51]. These findings suggest that maintaining protein homeostasis is crucial for proper neuronal function.

While SMA motor neurons have been thoroughly investigated, the knowledge about SMA muscles and their role in SMA pathology still remains elusive. It has been reported that SMA muscle cells show intrinsic defects in myogenic differentiation and energy metabolism [8, 9, 28] and that the proteome of skeletal muscles of presymptomatic SMA mice is dysregulated prior neuronal degeneration [50]. However, whether or how defects in muscles influence motor neuron physiology, and whether this contributes to SMA pathology is utterly unknown. Here, we report that SMA muscle cells differentially secrete proteins and that this contributes to patho-phenotypes of SMA motor neurons. Furthermore, we characterized the role of CTRP3, whose secretion and expression were reduced in SMA muscles, in motor neuron physiology.

## Materials and methods

### Animal model

Cells and tissues were isolated from an SMA mouse model [30]. Heterozygous *Smn* KO mice (*Smn*^KO/WT^;*SMN2*^0/0^) and human *SMN2* containing mice (*Smn*^KO/KO^;*SMN2*^tg/tg^) in congenic FVB/N background were bred to produce 50% of offspring as SMA mice (*Smn*^KO/KO^;*SMN2*^tg/0^) and 50% phenotypically normal heterozygotes (*Smn*^KO/WT^;*SMN2*^tg/0^) in each litter [59]. Wild type mice were used as controls (Jackson). Animal care and all additional procedures were performed according to the institutional animal care committee guidelines and the German animal welfare laws and approved under the reference numbers 84–02.05.20.13.042, 84–02.04.2015.A378 and UniKoeln_Anziege§4.16.020 and §4.17.025 of the LANUV (Landesamt für Natur, Umwelt und Verbraucherschutz NRW) state agency of North-Rhine-Westphalia.

### Recombinant proteins and drugs

Full-length recombinant proteins were purchased from Cusabio (mouse CTRP3: CSB-EP875360MO; human CTRP3: CSB-EP883621HU) and MyBioSource (human CTRP3: #MBS1265203). The water soluble mTOR inhibitor WYE-687 dihydrochloride was purchased from Tocris Bioscience (#4282) and the protein synthesis inhibitor anisomycin was purchased from Sigma-Aldrich (#A9789).

### Culture of cell lines and siRNA transfection

Muscle cell lines H-2K^b^-tsA58, C2C12, and motor neuron like cell line NSC-34 cells were cultured in Dulbecco’s Modified Eagle’s Medium (DMEM) with 10% fetal calf serum (Biochrom) and penicillin/streptomycin (Thermo Fisher Scientific). For proliferation, culture medium of H-2K^b^-tsA58 cells was further supplemented with 0.5% chicken embryo extract (Seralab) and 0.02% interferon γ (Roche) and cells were maintained at 33 °C with 10% CO_2_. To differentiate H-2K^b^-tsA58 cells, cells were cultured in DMEM with 5% horse serum (Biochrom) at 37 °C. C2C12 cells were differentiated in DMEM with 2% horse serum. For the differentiation of NSC-34 cells, growth medium was supplemented by 50 μM retinoic acid (Sigma) for 3 days. The knockdown of *Smn* in H-2K^b^-tsA58 and C2C12 cells was performed using siRNA (*Smn* #4390771 and negative control #4390843 Silencer Select Pre-Designed siRNA, Thermo Fisher Scientific) and Lipofectamine®2000 reagent (Thermo Fisher Scientific). Unless otherwise stated, all cells were maintained at 37 °C in a humidified incubator with 5% CO_2_.

### Primary motor neuron culture

Primary motor neurons were isolated from E13 embryos. After spinal cords were dissociated in 1% Trypsin (Worthington) with DNase I (Applichem) via triturating, cells were seeded on poly-D-lysine (PDL, Sigma) coated plates/coverslips with neuronal plating media (DMEM supplemented with 5% fetal calf serum (Biochrom), 0.6% glucose, penicillin/streptomycin (Thermo Fisher Scientific) and amphotericin B (Promocell)). 25,000 cells/cm^2^ were plated for imaging analyses and 145,000 cells/cm^2^ were plated for protein analyses. On the following day, neuronal plating media was replaced by motor neuron maintenance medium (Neurobasal medium supplemented with B27 supplement (Thermo Fisher Scientific), 2 mM L-glutamine, penicillin/streptomycin and amphotericin B with additional growth factors: ciliary neurotrophic factor (CNTF, 10 ng/ml, PeproTech), brain derived neurotrophic factor (BDNF, 10 ng/ml, PeproTech) and glia cell line derived neurotrophic factor (GDNF, 10 ng/ml, PeproTech). One-half of media was changed every 3rd day, and cytosine arabinoside (AraC) was added at 3DIV to a final concentration of 1 μM.

### RNA isolation, cDNA synthesis and real-time PCR

Total RNA was extracted from motor neurons using the mirVana™ miRNA Isolation Kit (Thermo Fisher Scientific). We followed the manufacturer’s instructions. RNA concentration was determined using the NanoDrop ND-1000 spectrophotometer (Peqlab). cDNA was produced from total RNA using the High-Capacity cDNA Reverse Transcription Kit (Thermo Fisher Scientific) with random primers. mRNA expression was determined by real-time PCR with PowerSYBR® Green PCR Master Mix (Thermo Fisher Scientific) and 1 μM of gene specific primers. The amplification conditions for *Actb* were: an initial incubation stage at 50 °C for 2 min, denaturation at 95 °C for 10 min and 40 cycles of amplification step (95 °C for 15 s, 60 °C for 30 s, and 72 °C for 40 s). The same conditions were used for the amplification of the other genes, except of different annealing temperatures (*Ctrp3*: 65 °C, *Smn*: 58 °C, *SMN2*: 59 °C, *Vegf*: 65 °C). An additional dissociation step was added to confirm the amplified product, and the PCR product was always confirmed by Sanger sequencing. Real-time PCR was performed with 7500 Real-Time PCR System (Thermo Fisher Scientific). Sequences of gene specific primers are listed in Additional file 1: Table S1.

### Protein isolation from cells, tissues, organs and blood plasma, and Western blot analysis

For plasma, tissue and organ isolation, P7 mice were sacrificed by decapitation and blood was collected from the open throat using EDTA-treated capillary blood collection tubes (Sarstedt). Blood cells were eliminated by centrifugation. Proteins of cultured cells, tissues and organs were extracted with RIPA buffer (Sigma) supplemented with protease and phosphatase inhibitors (Thermo Fisher Scientific). Snap frozen tissues and organs were homogenized using ceramic beads in a Precellys24 device (Peqlab) and DNA was sheared by sonication. Protein concentration was determined by BCA assay (Thermo Fisher Scientific). Equal protein amounts were confirmed by SDS–PAGE and Western blot analysis with house-keeping proteins and/or Ponceau staining. The information about antibodies are listed in Additional file 1: Table S8. Signals were detected with ChemiDoc XRS + System (BioRad), and quantification of signals were performed using ImageLab software (BioRad).

### Proteomics of muscle cell proteomes and secretomes

To screen for differentially expressed and secreted proteins of control and SMN-deficient H-2K^b^-tsA58 myotubes, we followed a previously published protocol with some modifications [20]. In brief, cells were transfected with siRNA and simultaneously, differentiation was induced in SILAC DMEM supplemented with azidohomoalanine (AHA) and stable isotopes of arginine and lysine (control: ^13^C_6_-Arg, d_4_-Lys; *Smn*KD: ^13^C_6_^15^N_4_-Arg, ^13^C_6_^15^N_2_-Lys). After 72 h, the S*mn* knockdown (KD) efficiency was verified by Western blot analysis, and cell lysates/conditioned media of control and *Smn* KD myotubes were combined. The conditioned medium was concentrated using 3 kDa cutoff filters (Millipore) and newly synthesized proteins from cells and secretomes (concentrated medium) were enriched using the Click-iT Protein Enrichment Kit (Thermo Fisher Scientific) following the manufacturer’s protocol. Resin-bound proteins were reduced using 10 mM DTT, alkylated with 50 mM acrylamide, and finally digested with trypsin overnight at 37 °C. Peptide solutions were desalted using Oasis HLB 1 cc Cartridges (Waters) and fractionated in 12 fractions by OFFGEL electrophoresis using 13 cm pI 3–10 strips (GE Healthcare) as described elsewhere [64]. Samples were purified using C18 STAGETips [58], dried using a vacuum centrifuge, and resuspended in 5% formic acid 5% acetonitrile. Samples were analyzed by LC-MSMS using an EASYnLC1000 in combination with an Orbitrap Velos (both Thermofisher Scientific) using 60 min linear gradients from 100% solvent A (water with 0.1% formic acid) to 35% solvent B (acetonitrile with 0.1% formic acid) 65% solvent A (for details see, [64]). Raw data were analyzed using MaxQuant software 1.5.3.8 [14] with the following parameters: variable modifications: replacement of methionine by AHA, acetylation at protein N-termini, oxidation at methionine; fixed modification: propionamide at cysteine; maximum number of modifications: 5; quantification multiplicity: 3plex SILAC; missed cleavage sites:2; MS mass tolerance first pass search: 20 ppm; MSMS mass tolerance: 0.5 Da. Match between runs was activated and the database used was Uniprot mouse (release date 22.07.2015) in combination with common contaminants.

### Proteomics of neuronal cells

Differentiated NSC-34 cells were treated with 5 μg/ml recombinant human CTRP3 protein for 6 h. Cells were lysed in RIPA buffer with protease and phosphatase inhibitors and DNA was sheared by sonication. Proteins were precipitated using ice-cold acetone and the pellet was dissolved in 6 M urea/ 2 M thiourea. This was followed by an in-solution reduction using 5 mM dithiothreitol (DTT), an alkylation using 40 mM iodoacetamide (IAA) and the digestion with endoproteinase Lys-C and trypsin. Acidified peptide samples were purified using styrenedivinylbenzene-reverse phase sulfonate (SDB-RPS) Stage Tips [58]. An Easy nLC 1000 ultra-hig performance liquid chromatography (UHPLC) coupled to a QExactive Plus Hybrid Quadrupole-Orbitrap mass spectrometer (Thermo Scientific) was used for proteomic analysis. Raw data were analyzed using the Andromeda search engine (MaxQuant software 1.5.3.8) [14]. Parameters in MaxQuant were set to default with trypsin selected as protease for digestion. Additionally, match-between runs and LFQ quantification algorithms were enabled. A mouse database from Uniprot (16.06.17) with contaminants was used for peptide and protein identification. Statistical analysis, GO annotations, t-tests and data visualization were performed using the Perseus software [71] and the Instant Clue software [53].

### SUnSET assay (SUrface SEnsing of translation)

To monitor the effect of CTRP3 on the protein synthesis efficiency, we performed SUnSET assay [63]. In brief, culture medium of differentiated NSC-34 cells was treated with 5 μg/ml recombinant mouse CTRP3 protein for either 2 or 6 h. 1 h prior protein collection 1 μM puromycin was added to label proteins that are in the middle of synthesis. To measure the protein synthesis efficiency, puromycin-labelled proteins/peptides were detected by Western blot analysis using anti-puromycin antibody. Protein synthesis efficiency was compared with non-treated control cells.

To measure protein synthesis in primary motor neurons, 3DIV motor neurons were treated with 5 μg/ml recombinant human CTRP3 for 2 h and proteins were labelled with 1 μM puromycin for 30 min. After that, neurons were fixed with 4% paraformaldehyde (PFA) for 20 min, and puromycin signals were visualized with anti-puromycin antibody (modified from [44]). Images were taken blindly with a light microscope (Zeiss) and analysed with Fiji.

### Axon outgrowth assay

25,000 motor neurons/cm^2^ were seeded on PDL-coated cover-slips and grown for 3 days. Two days after plating, motor neurons were treated with 5 μg/ml recombinant human CTRP3 for 24 h, and cells were fixed with 4% PFA for 20 min. Neuronal morphology was visualized with anti-TAU antibody and motor neuron identity was confirmed with anti-ChAT antibody. Images were taken blindly with a light microscope (Zeiss) and analysed with Fiji. The information about antibodies can be found in Additional file 1: Table S2**.**

### Image analysis

All images were acquired with a light microscope (Zeiss Axio Imager.M2) equipped with an AxioCam MR camera and an ApoTome.2 system (Institute of Human Genetics, University of Cologne). Images were analyzed with the ZEN (Zeiss) or Fiji. All image analyses were performed blindly.

### Statistical test and GO analysis

Statistical tests were performed by Prism (Graphpad) and GO/pathway analysis was performed by g:Profiler and Reactome (reactome.org).

## Results

### Systemic muscle secretome analysis: secretion of CTRP3 is reduced in SMN-deficient muscle cells

**Fig. 1:**
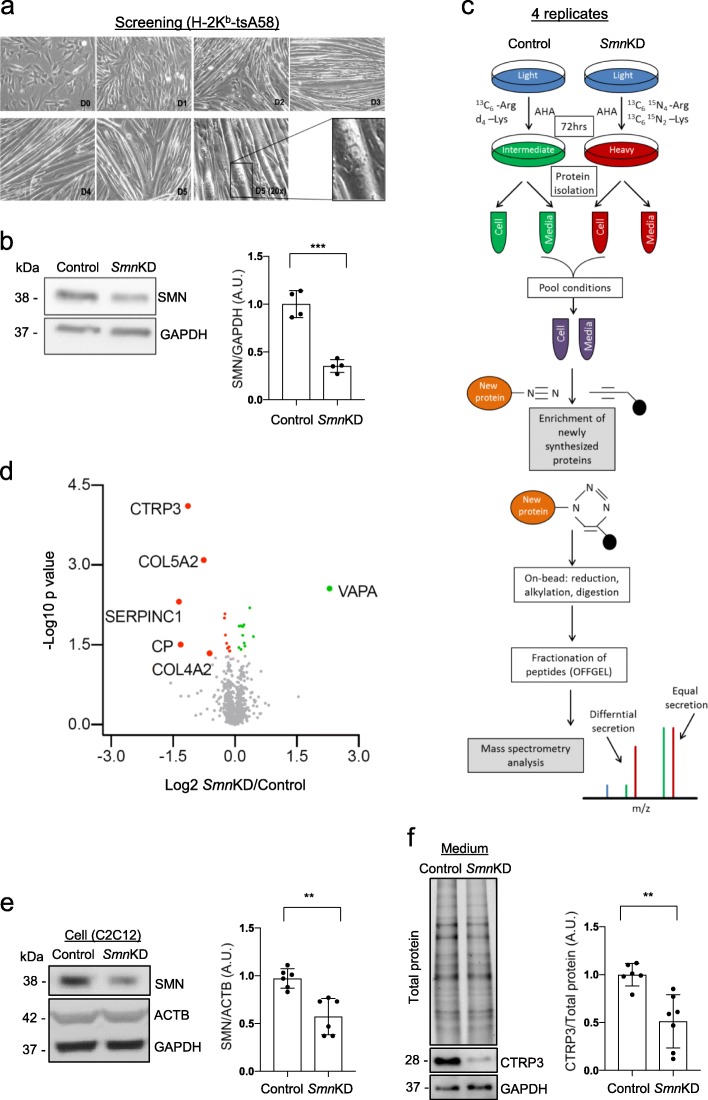
SMN-depleted muscle cells secrete reduced levels of CTRP3 protein. Quantitative secretome analysis of metabolically labelled SMN-depleted muscle cells. **a** H-2K^b^-tsA58 cells were differentiated to multinucleated myotubes within 3 to 5 days (10x, 20x). **b** Representative Western blots and quantification showing knockdown efficiency. Negative control siRNA (Control) or siRNA against *Smn* (SmnKD) was transfected, and cells were differentiated for 72 h. GAPDH was used as a loading control (*n* = 4). **c** Schematic drawing of quantitative secretome analysis. Simultaneous siRNA transfection and combined pulsed labelling of differentiating H-2K^b^-tsA58 muscle cells with azidohomoalanine (AHA) and stable isotope-labelled amino acids. Newly synthesized proteins were isolated by click chemistry and analyzed by mass spectrometry. **d** Volcano plot of secretome analysis; statistical significance (−log10, *p*-value) against fold change (FC, log2, Control/SmnKD). Normalized SILAC ratios of 4 replicates were used to calculate the log2 ratio between SmnKD (heavy) and control (intermediate) conditions, and *p*-values were determined using an unpaired two-sided t-test. Proteins with a *p*-value < 0.05 are highlighted in green/red and proteins with a FC > 50% are additionally labelled with name. **e** Representative Western blots and quantification of C2C12 cells transfected with negative control siRNA (Control) or siRNA against *Smn* (SmnKD), and differentiated for 5 days. ACTB was used as a loding control. **f** Representative Western blots and quantification of serum-free conditioned media of control and *Smn* KD C2C12 cells, differentiated for 5 days. CTRP3 levels were normalized to the total protein amount (stainfree gel). Dot graphs represent data from independent experiments and the bar graphs depict the mean ± s.d. Two-tailed unpaired student’s t-test was used to determine statistical signaficance. ***p < 0.001; **p < 0.01

We screened secreted proteins from control and SMN-deficient muscle cells with a combination of two metabolic labeling methods and mass spectrometry (MS). As muscle cells need 5% of horse serum for differentiation and horse serum contains numerous proteins, we adapted Click-iT AHA (L-azidohomoalanine) method to distinguish muscle secreted proteins from serum proteins in conditioned culture media. Additionally, we also used SILAC (stable isotope labeling by amino acids in cell culture) [54] to determine whether proteins are secreted from control cells or SMA cells. Immortalized H-2K^b^-tsA58 (H-2K^b^) myoblasts were cultured and differentiated as previously described [49]. In brief, cells fused and formed myotubes within 3 days in differentiation medium (Fig. 1a). With siRNA technology and transient transfection, we could successfully reduce SMN protein levels to ~ 40% of control levels in 72 h (Fig. 1b). With this optimized protocol, cells were cultured in 500 μM AHA containing SILAC differentiation media, and the medium of control cells (negative control siRNA, siCon) was supplemented with intermediate isoforms of arginine and lysine (^13^C_6_-Arg, d_4_-Lys) and *Smn* KD (*Smn* knockdown, siSmn) with heavy isoforms (^13^C_6_^15^N_4_-Arg, ^13^C_6_^15^N_2_-Lys) for 72 h. Next, we collected media from control and *Smn* KD muscle cells, enriched and isolated newly synthesized proteins with Click-iT chemistry. With this condition, we screened secreted proteomes from control and *Smn* KD muscle cells with mass spectrometry (Fig. 1c). Importantly, we have confirmed that 72 h treatment of 500 μM AHA has neither an effect on muscle cell differentiation nor the cell death (Additional file 2: Figure S1). From 4 replicated experiments, we have detected 1739 proteins from whole secretomes, and among them, levels of 25 proteins were significantly altered by SMN deficiency (*p*-value < 0.05, Fig. 1d, Table 1 and Additional file 3: Data S1). Pathway analysis has revealed that muscle-secreted proteins regulate eukaryotic translation and SLIT/ROBO expression, which are important for axon guidance and motor neuron positioning in spinal cords [36, 43] (Additional file 1: Table S3). Furthermore, we also performed pathway analysis of differentially secreted proteins in *Smn* KD muscle cells. Interestingly, collagen metabolism and the regulation of IGF (insulin like growth factor) pathway are significantly altered in *Smn* KD muscle secretomes (Additional file 1: Table S4**)**. As collagen metabolism is important for the function of extracellular matrix at the neuromuscular junction [39, 62] and IGF pathway has been implicated in SMA pathology [5, 70], this finding substantiates the quality of our data.

**Table 1:** Differentially secreted proteins from control and *Smn* KD H-2K^b^-BL6 muscle cells (*p* < 0.05)

Gene name	Protein name	# of unique peptides	*P* value	Fold change *Smn*KD/Cont	GO/KEGG
*Vapa*	Vesicle-associated membrane protein-associated protein A	2	0.0028	4.903	Tight junction
*Cxcl1*	Fractalkine	3	0.0220	1.365	Chemokine signaling exosome
*Dpp2*	Dipeptidyl peptidase 2	5	0.0063	1.286	exosome
*Serpinh1*	Serpin H1	15	0.0331	1.182	
*Timp1*	Metalloproteinase inhibitor 1	6	0.0293	1.167	Exosome
*Alad*	Delta-aminolevulinic acid dehydratase	7	0.0132	1.165	
*Col4a1*	Arresten;Collagen alpha-1(IV) chain	33	0.0209	1.140	Extracellular matrix, exosome
*Lgals1*	Galectin-1	8	0.0142	1.139	exosome
*Serpine2*	Glia-derived nexin	9	0.0386	1.109	exosome
*Rcn1*	Reticulocalbin-1	6	0.0138	1.101	Ca2+ binding
*Msn*	Moesin	23	0.0140	1.071	Actin cytoskeleton, cell junction, exosome
*Lgal3bp*	Galectin-3-binding protein	5	0.0353	1.070	exosome
*Lsyna1*	Inositol-3-phosphate synthase 1	7	0.0411	0.920	
*Atp5j2*	ATP synthase subunit f, mitochondrial	1	0.0414	0.914	ATP synthesis, oxidative `phosphorylation
*Tbl1xr1*	F-box-like/WD repeat-containing protein TBL1XR1	2	0.0346	0.907	Wnt pathway,
*Actn2*	Alpha-actinin-2	22	0.0364	0.885	Cell junction, actin cytoskeleton
*Xirp1*	Xin actin-binding repeat-containing protein 1	4	0.0295	0.871	Cell junction, actin cytoskeleton
*Uqcrc1*	Cytochrome b-c1 complex subunit 1, mitochondrial	7	0.0206	0.852	Oxidative phosphorylation, Alzheimer’s, Parkinson’s, Huntington diseases
*Phb2*	Prohibitin-2	7	0.0082	0.845	
*Ermp1*	Endoplasmic reticulum metallopeptidase 1	2	0.0099	0.841	
*Col4a2*	Canstatin; Collagen alpha-2(IV) chain	36	0.0454	0.655	Focal adhesion, exosome
*Col5a2*	Collagen alpha-2(V) chain	37	0.0008	0.592	Focal adhesion, exosome
*C1qtnf3*	Complement C1q tumor necrosis factor-related protein 3	3	7.77E-0.5	0.455	Glucose homeostasis, exosome
*Cp*	Ceruloplasmin	2	0.0313	0.402	Cu-oxidase, exosome
*Serpinc1*	Antithrombin-III	5	0.0049	0.391	Complement and coagulation cascades, endopeptidase inhibitor, exosome

We have also performed same experiments with muscle cell samples. Notably, with ~ 40% of SMN levels, only 30 proteins were significantly altered using the same criteria for secretome analysis (Additional file 2: Figure S2 and Additional file 1: Table S5). Consistent with the secretome data, SMN deficient muscle cells also showed altered protein levels linked to collagen metabolism, protein and RNA homeostasis and peroxisomal protein import (Additional file 1: Table S6).

Among 25 differentially secreted proteins, the most significantly altered protein was C1QTNF3 (C1q/tumor necrosis factor-related protein 3, also known as CTRP3, Fig. 1d). We tested whether CTRP3 secretion is also changed in another muscle cell line, C2C12. We knocked down *Smn* with siRNA, differentiated cells and measured secreted CTRP3 levels in conditioned media by Western blot analysis. Indeed, with ~ 50% reduction of SMN, CTRP3 secretion is reduced in *Smn* KD C2C12 cells (Fig. 1e and f). From these findings, we concluded that proteins are differentially secreted from SMA muscle cells and that CTRP3 is the most strongly changed secreted protein upon SMN depletion.

### CTRP3 levels are reduced in *tibialis anterior* muscle tissues and plasma of SMA mice

**Fig. 2:**
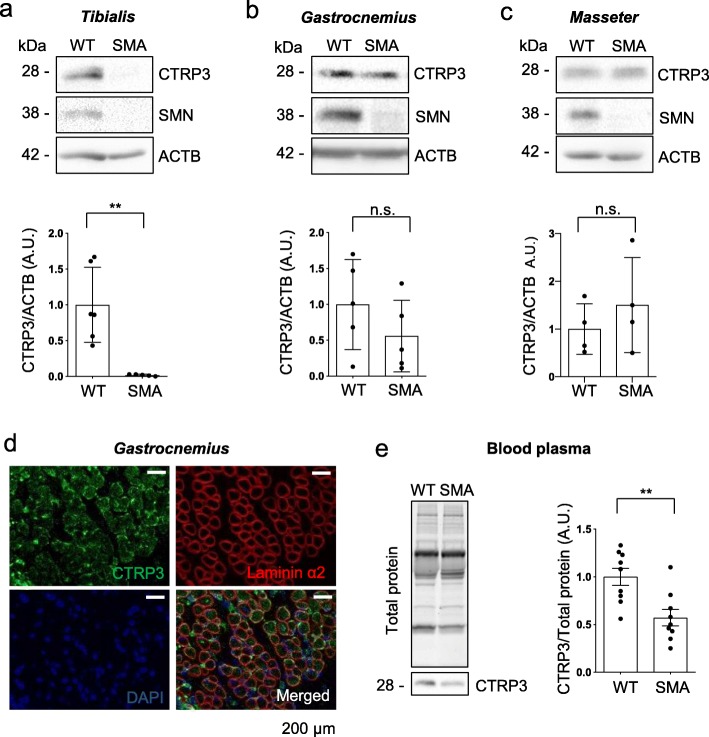
CTRP3 levels are reduced in *tibialis anterior* muscle and blood plasma of SMA mice. Representative Western blots and quantification of CTRP3 levels in SMA mice. **a** *tibialis anterior* (WT *N* = 6; SMA *N* = 5), **b** *gastrocnemius* (*N* = 5) and **c** *masseter* (*N* = 4) muscles of P7 WT and SMA mice. ACTB was used as a loading control. **d** Immunofluorescence staining of an cross section of *gastrocnemius* muscle of a P7 WT mouse, using anti-Laminin α2 (red), anti-CTRP3 (green) and DAPI (blue). Scale bars = 200 μm. **e** Representative Western blots and quantification of blood plasma proteins isolated from P7 WT and SMA mice. CTRP3 levels were normalized to the total protein amount (stainfree gel) (*N* = 9). Dot graphs represent data from independent experiments and the bar graphs depict the mean ± s.d. Two-tailed unpaired student’s t-test was used to determine statistical signaficance. ** *p* < 0.01, n.s = not significant, *p* > 0.05

Next, we assessed CTRP3 levels in an SMA mouse model [30]. We measured the levels of CTRP3 in three different muscle tissues from postnatal day 7 (P7) WT and SMA mice. Interestingly, CTRP3 levels were exceedingly reduced in *tibialis anterior*, while they were not significantly changed in *gastrocnemius* and *masseter* muscles (Fig. 2a-c). These data are in line with previous reports suggesting that SMN deficiency influences gene expression in muscle [8, 9] and *tibialis anterior* has been reported as a vulnerable muscle tissue in the same SMA mouse model used in this study [67]. Next, we checked the localization of CTRP3 proteins in muscle tissues. In gastrocnemius muscle of P7 mice, CTRP3 protein signal was detected in muscle fibers as dot-like structures and it seems enriched in the extracellular matrix (Fig. 2d and Additional file 2: Figure S3). Furthermore, as CTRP3 is a secreted molecule, we tested CTRP3 levels in blood plasma from P7 WT and SMA mice and found that blood plasma CTRP3 levels are significantly reduced in SMA mice (Fig. 2e). In addition, as liver is the major source of blood plasma proteins, we measured the levels of CTRP3 in liver. CTRP3 levels in livers of P7 mice seem unaltered by SMA (Additional file 2: Figure S4). Taken together, we confirmed that CTRP3 levels were reduced in *tibialis anterior* muscle and plasma of SMA mice.

### CTRP3 regulates protein synthesis in neurons

As secretion of CTRP3 is reduced in SMA muscles, we further investigated the role of CTRP3 in motor neuron physiology. Originally, CTRP3 has been described as an adipokine, which is important for metabolism related pathways in various tissues [78]. In brain, it plays a neuroprotective role after injury caused by intracerebral hemorrhage [73]. Therefore, we first measured CTRP3 levels in brain and spinal cord of WT and SMA mice. CTRP3 proteins were detected in both brain and spinal cord from P7 WT and SMA mice, but the levels of CTRP3 were reduced only in brain of SMA mice (Additional file 2: Figure S5). Prior to analyzing downstream pathways, we optimized exogenous CTRP3 treatment in motor neurons. For optimization, we used a motor neuron-like cell line, NSC-34 cells. NSC-34 cells were differentiated 3 days with 50 μM retinoic acid (RA) and treated with recombinant CTRP3 protein (Additional file 2: Figure S6). We found that 5 μg/ml CTRP3 induces changes in signaling pathways in NSC-34 cells. For further experiments, we used 5 μg/ml as optimal condition.

**Fig. 3:**
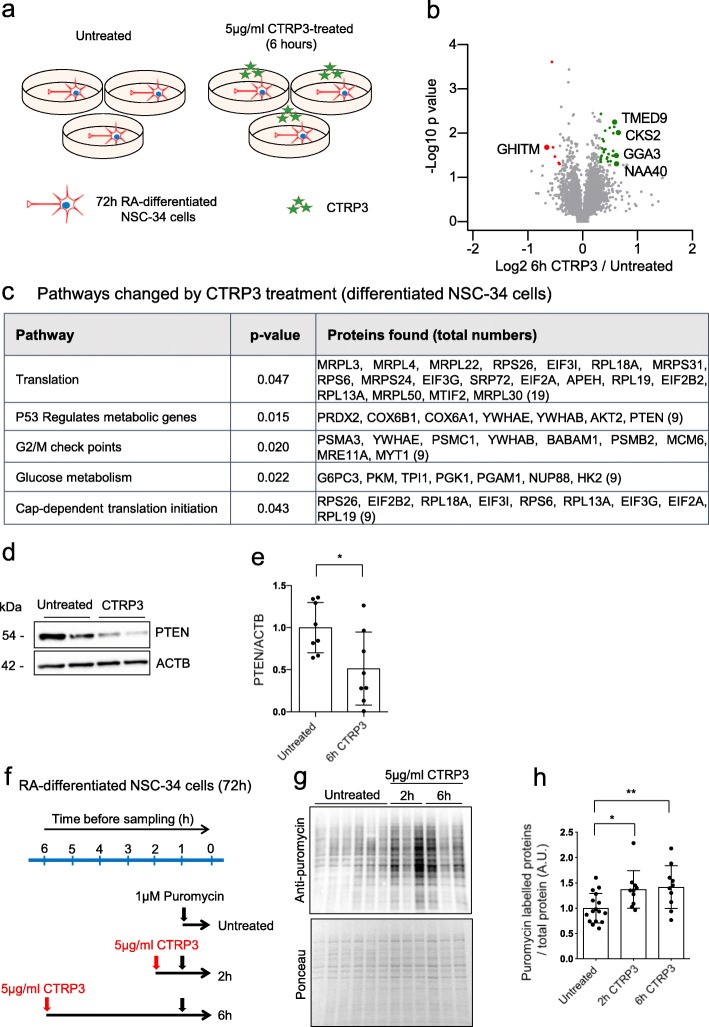
CTRP3 treatment stimulates protein synthesis in motor neuron-like NSC-34 cells. Downstream pathway analysis of CTRP3 in motor neuron-like NSC-34 cells. **a** Schematic drawing showing whole proteome analysis of differentiated NSC-34 cells. **b** Volcano plot of whole proteome analysis; plotted statistical significance (−log10, p-value) against fold change (log2, CTRP3 treated/untreated). Cells were treated with 5 μg/ml recombinant mouse CTRP3 (mCTRP3). Three independent samples were used for analysis. *P*-values were determined using an unpaired two-sided t-test (*n* = 3). Proteins with *p* < 0.05 are highlighted in green (upregulated)/ red (downregulated) and proteins with *p* < 0.05 and FC > 50% are additionally labelled with name. **c** Representative pathways changed by CTRP3 treatment. (proteins with *p* < 0.1 and pathways with *p* < 0.05, with g:Profiler). **d** Representative Western blots for PTEN and **e** quantification of PTEN levels in CTRP3 treated differentiated NSC-34 cells, Two-tailed unpaired student’s t-test was used to determine statistical signaficance, **f** Scheme of SUnSET assay. Differentiated NSC-34 cells were treated with mCTRP3 for 2 or 6 h. Cells were incubated with 1 μM puromycin for 1 h before analysis. **g** SUnSET assay shows that CTRP3 increases protein synthesis. **h** Dot plot bar graph summarizes SUnSET assay. SUnSET data were normalized to the total protein amount (ponceau). Each dot represents an independent experiment and the bar graph represents mean ± s.d. (*n* = 16/10/10). One way ANOVA with Fisher’s LSD test was used to determine statistical significance; * *p* < 0.05, ** *p* < 0.01

To obtain a systemic view of CTRP3 function in neurons, we next profiled downstream pathways of CTRP3 by mass spectrometry. We differentiated NSC-34 cells with RA for 3 days, treated them with 5 μg/ml CTRP3 for 6 h, and performed whole proteome analysis (Fig. 3a and Additional file 4: Data S2). From the profiling, 5653 proteins were detected, and 118 proteins were significantly altered (*p* < 0.05). However, levels of only 33 proteins were changed more than 25% by CTRP3 treatment. Among them, the most upregulated protein was CKS2 (cyclin-dependent kinases regulatory subunit 2), and the most downregulated protein was GHITM (growth hormone inducible transmembrane protein) (Fig. 3b and Additional file 1: Table S7). As the number of altered proteins with these criteria was too low, we used proteins with *p* < 0.1 for pathway analysis, which are 291 proteins. Thereby, we found that pathways associated with protein synthesis including translation initiation, elongation, ribosomes and insulin receptor recycling are predominantly altered by CTRP3 treatment (Fig. 3c and Additional file 1: Table S8**)**. Interestingly, PTEN expression was reduced about ~ 50% by CTRP3 treatment. As PTEN plays an important role in protein synthesis, we confirmed the PTEN levels in CTRP3 treated NSC-34 cells by Western blot analysis (Fig. 3d and e). This data validates quality of our mass spectrometry experiment. Next, we biochemically measured the protein synthesis efficiency in CTRP3 treated NSC-34 cells with SUnSET (SUrface SEnsing of Translation) method. In brief, control and CTRP3 treated NSC-34 cells were incubated with a very low concentration of puromycin (1 μM) for 1 h. Puromycin labelled the proteins synthesized during the given time, and these proteins could be detected with an antibody against puromycin [63]. The intensity of puromycin signal, in other word, the amount of puromycin labelled newly synthesized proteins was used as a measure for the protein synthesis rate. We treated CTRP3 to NSC-34 cells for 2 or 6 h and measured the protein synthesis efficiency (Fig. 3f). Indeed, we confirmed that CTRP3 enhanced the protein synthesis rate in motor neuron like NSC-34 cells (Fig. 3g and h).

### CTRP3 enhances SMN and VEGF levels in motor neurons

**Fig. 4:**
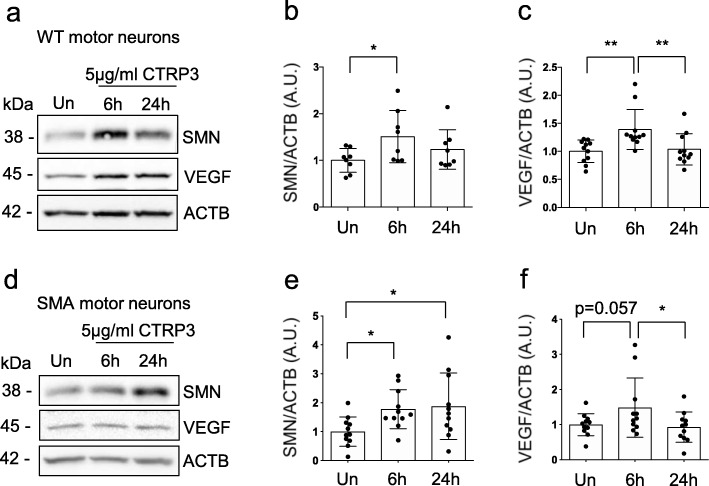
CTRP3 increases SMN and VEGF protein levels in primary WT and SMA motor neurons. Representative Western blots and quantification of SMN and VEGF protein levels in CTRP3 treated motor neurons isolated from spinal cord of E13 mice. Embryonic motor neurons from WT (**a**-**c**) and SMA (**d**-**f**) mice were cultured for 5 days in vitro (5DIV) and treated with 5 μg/ml mCTRP3 protein for 6 or 24 h. **b**, **c**, **e** and **f** Dot plot bar graphs represent quantification of VEGF and SMN levels. ACTB was used as a loading control. Each dot represents independent experiment and the bar graph represents mean ± s.d. (WT-SMN: *n* = 11, *N* = 5; WT-VEGF: *n* = 7, *N* = 3; SMA-SMN: *n* = 8; *N* = 3; SMA-VEGF: *n* = 11, *N* = 5, n: number of independent experiments, N: number of independent neuron cultures) One way ANOVA with Fisher’s LSD test was used to determine statistical significance; * *p* < 0.05, ** *p* < 0.01

While SMN is the most important protein for SMA, we could not detect the SMN protein with mass spectrometric analysis of CTRP3 treated NSC-34 cells. Therefore, we measured SMN protein levels in motor neurons after CTRP3 treatment. Additionally, it has been reported that CTRP3 regulates VEGF levels in brain [73] and that extremely severely affected SMA patients and mice show peripheral necrosis, which can be caused by vascular defects [2, 30]. Therefore, we also independently measured VEGF protein levels in motor neurons after CTRP3 treatment. We found that both SMN and VEGF protein levels were elevated in WT and SMA motor neurons upon CTRP3 treatment (Fig. 4a-f). It is worthy to note that SMA neurons showed rather slow increase in SMN protein levels after CTRP3 treatment compared to WT ones. In WT motor neurons, CTRP3 treatment increases SMN levels in 6 h the most, but in SMA motor neurons, SMN levels continue to increase in 24 h (Fig. 4d and e). While we have already shown that CTRP3 increases the efficiency of protein synthesis in neurons, mRNA levels were also measured by quantitative RT-PCR to clarify the molecular mechanism underlying elevation of SMN and VEGF protein levels. Indeed, neither of mRNA levels was altered (Additional file 2: Figure S7). These results indicate that elevated SMN and VEGF levels are due to the enhanced translation. From these findings, we concluded that CTRP3 enhances protein synthesis including SMN and VEGF in neurons. Subsequently, we investigated the cellular mechanisms underlying CTRP3-mediated protein synthesis.

### CTRP3 induced SMN protein synthesis is mTOR dependent

**Fig. 5:**
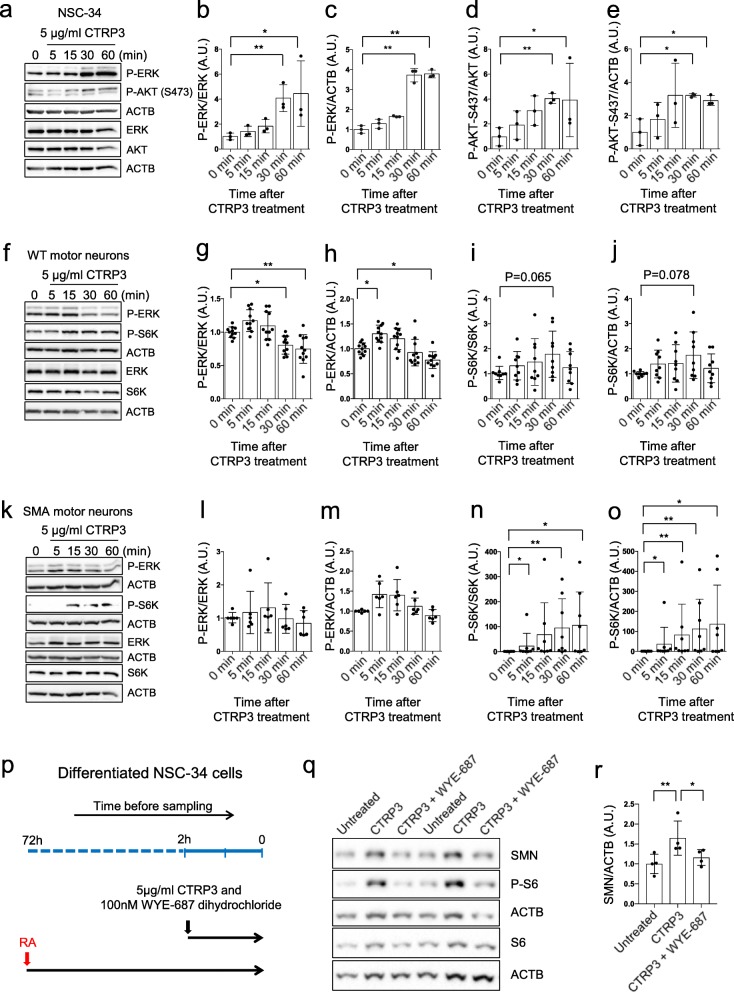
CTRP3 activates the PI3K/mTOR and MAPK/ERK pathway in motor neurons. **a**-**e** Representative Western blots and quantification of AKT and ERK activity in NSC-34 cells treated with 5 μg/ml mCTRP3 for 5, 15, 30 or 60 min. Activity of PI3K and MAPK/ERK pathways were measured using anti-p-ERK (Thr202/Tyr204) and anti-p-AKT (S473) antibodies. (*n* = 3) **f**-**j** WT and **k**-**o** SMA motor neurons treated with 5 μg/ml mCTRP3 for 5, 15, 30 or 60 min. Activity of PI3K/mTOR and MAPK/ERK pathways were measured using anti-p-S6K (Thr389) and anti-p-ERK (Thr202/Tyr204) antibodies. ACTB was used as a loading control. **g**-**j**, **l**-**o** Dot plot bar graphs summarize the repeated experiments. Each dot represents an independent experiment and bar graphs depict the mean ± s.d. (WT: *n* = 11 for p-ERK and *n* = 9 for p-S6K, SMA: *n* = 6 for p-ERK *n* = 8 for p-S6K) Kruskal Wallis and Uncorrected Dunn’s test was used to determine statistical significance; ** *p* < 0.01 and * *p* < 0.05. **p** Differentiated NSC-34 cells were treated with 100 nM of water-soluble mTOR inhibitor WYE-687 dihydrochloride and/or 5 μg/ml human CTRP3 (hCTRP3) for 2 h. **q** Representative Western blots of NSC-34 cells with anti-p-S6 (Ser235/236), anti-S6 and anti-SMN antibodies show that elevated SMN levels by CTRP3 treatment were mTOR dependent. ACTB was used as a loading control. **r** Dot plot bar graphs summarize the repeated experiments. Each dot represents an independent experiment and bar graphs depict the mean ± s.d. Kruskal Wallis and Uncorrected Dunn’s test was used to determine statistical significance; ** *p* < 0.01 and * *p* < 0.05

While there are various mechanisms regulating protein synthesis, best known signaling pathways regulating mRNA translation in neurons are the PI3K/mTOR and MAPK/ERK pathways. Of note, SMA neurons showed reduced mTOR activity and impaired protein synthesis [37], as well as dysregulated MAPK/ERK pathway [4]. Therefore, we tested whether CTRP3 modulates these two pathways in NSC-34 cells as well as WT and SMA primary motor neurons. First, we treated differentiated NSC-34 cells with 5 μg/ml CTRP3 and measured the phosphorylation state of ERK and AKT as a marker of active MAPK/ERK and PI3K pathway respectively (Fig. 5a-e). We found that the phosphorylation state of both ERK and AKT were enhanced upon CTRP3 treatment. It is noteworthy to mention that the activity of a direct target of mTOR kinase, S6K (p70S6K) was extremely low in NSC-34 cells, at least in our hands (data not shown). Therefore, we could not reliably quantify phosphorylation status of S6K in NSC-34 cells.

Next, we tested the effect of CTRP3 in 5DIV (days in vitro) primary WT and SMA motor neurons. In primary motor neurons, we could measure mTOR activity with phosphorylation status of S6K as well as ERK activity. In WT motor neurons, CTRP3 enhanced ERK activity moderately but significantly, while mTOR activity (p-S6K) was not significantly altered (Fig. 5f-j). To our surprise, in SMA motor neurons, CTRP3 increased mTOR activity enormously (Fig. 5k, n and o). As we have previously reported, SMA neurons often show extremely low basal mTOR activity [37], therefore, fold induction of S6K activity was more exaggerated in SMA motor neurons. While there was a trend of elevation, ERK activity was not significantly altered by CTRP3 treatment in SMA motor neurons (Fig. 5k, l and m).

mTOR kinases form two types of complexes based on binding partners: mTORC1 and mTORC2. They play distinct roles in cellular processes [27]. It has been known that mTORC1 mainly regulates protein synthesis, however, mTORC2 can also bind ribosomes directly [81]. mTORC1 phosphorylates p70S6K directly and mTORC2 phosphorylates AKT at S473. Therefore, these phosphorylation sites are often used as markers of mTORCs activity. To further characterize CTRP3-mediated mTOR activation, we also measured the phosphorylation status of AKT at S473 and found that CTRP3 does not activate mTORC2 in WT primary motor neurons (Additional file 2: Figure S8). This data suggest that CTRP3 enhances mTORC1 activity in primary motor neurons. Furthermore, we checked whether CTRP3 can regulate cap-dependent translation by phosphorylating 4E-BP1. Indeed, phosphorylation of 4E-BP1 was elevated by CTRP3 in WT motor neurons (Additional file 2: Figure S9). This finding strongly suggests that CTRP3 enhances protein synthesis via mTORC1, S6K and 4E-BP signaling cascade in motor neurons.

Finally, to validate the role of the mTOR pathway in CTRP3-induced SMN protein synthesis, we pharmacologically blocked mTOR activity and checked CTRP3-induced SMN protein synthesis in NSC-34 cells (Fig. 5p). Interestingly, in our hands, DMSO slightly but consistently increased the SMN protein level in motor neurons (data not shown). Therefore, we used the water-soluble mTOR inhibitor WYE-687 dihydrochloride to avoid DMSO as a vehicle. Indeed, WYE-687 dihydrochloride successfully diminished the CTRP3 induced SMN protein synthesis as well as mTORC1 activity (Fig. 5q, r, and Additional file 2: Figure S10). Additionally, we checked whether ERK pathway also contribute to the CTRP3-mediated SMN protein synthesis. To answer this question, we treated 20 μM ERK inhibitor U0126 together with CTRP3 to primary WT motor neurons, and checked whether U0126 can block CTRP3-mediated SMN protein synthesis. ERK inhibition does not affect CTRP3 mediated SMN level elevation (Additional file 2: Figure S11). Taken together, CTRP3 positively regulates protein synthesis including SMN via the mTORC1 pathway in motor neurons.

### CTRP3 influences motor neuron growth and axonal local protein synthesis

**Fig. 6:**
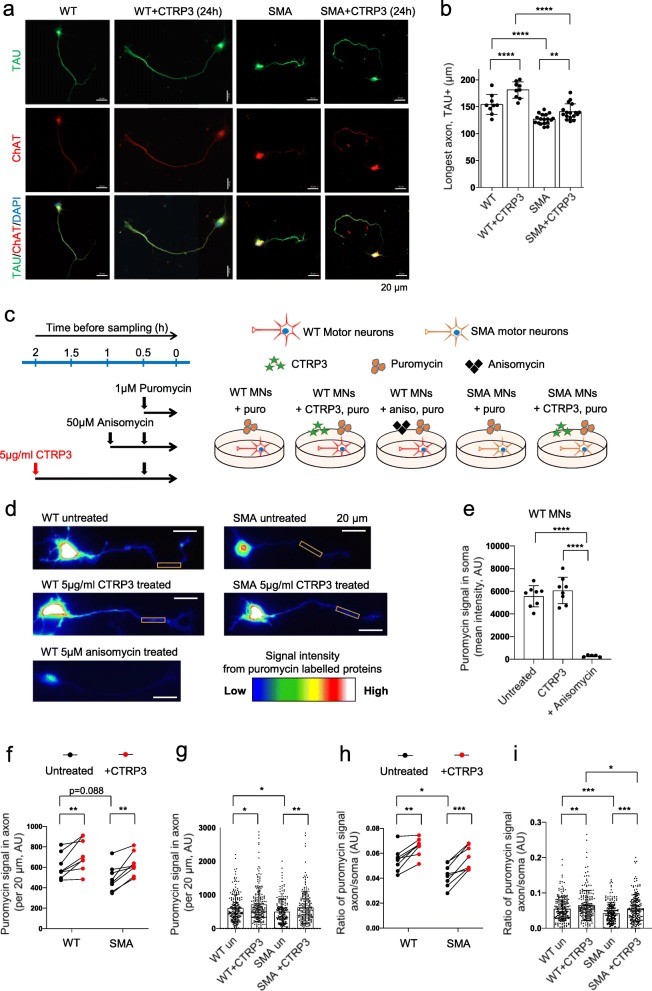
CTRP3 stimulates axonal growth and protein synthesis of motor neurons. **a** Axon outgrowth assay with WT and SMA motor neurons treated with 5 μg/ml hCTRP3 for 24 h (2DIV-3DIV). Representative images of motor neurons stained with anti-Tau (green), anti-ChAT (red) and DAPI (blue). Scale bars: 20 μm. **b** Dot plot bar graph summarizes axon outgrowth analysis. Each dot represents the average axon length of 20–25 neurons in a coverslip. Bar graphs depict the mean ± s.d. (WT: *n* = 9, *N* = 3; SMA: *n* = 17, *N* = 4; n = number of coverslips, N = number of independent cultures). Two way ANOVA with Sidak’s multiple comparison test was used to determine statistical significance; **p < 0.01, ****p < 0.0001. **c** Scheme of SUnSET assay to investigate the effect of CTRP3 on axonal protein synthesis. Motor neurons (3DIV) were treated with 5 μg/ml hCTRP3 for 2 h and newly synthesized proteins were labelled with 1 μM puromycin for 30 min before analysis. Data are compared with non-CTRP3 treated cells. As a negative control, protein synthesis was blocked with 50 μM anisomycin for 1 h before analysis. **d** Representative images of WT and SMA motor neurons immunologically stained with anti-puromycin antibody. Rainbow scale heatmap indicating the intensity of SUnSET signals. **e** Dot plot bar graph represents the mean intensity of the SUnSET signal in soma of WT motor neurons. Each dot represents the average value of 24–28 neurons from an independent culture and bar graphs depict the mean ± s.d. (Untreated n = 8/*N* = 8, CTRP3 treated *n* = 8/*N* = 8, anisomycin treated *n* = 5/*N* = 5, n = number of coverslips, N = number of independent cultures, 25 neurons per coverslip). Ordinary one-way ANOVA and Uncorrected Fisher’s LSD were used to determine statistical significance. ****p < 0.0001, **f**-**g** Quantification of the mean intensity of the SUnSET signal in axons (per 20 μm). **f** SUnSET signal in axon (WT *N* = 8, SMA *N* = 8, N = number of independent cultures). Each dot represents average data from one independent neuron culture. Therefore, data were only compared in the same culture with and without CTRP3 treatment. Two-tailed paired student’s t-test was used to determine statistical significance, **p < 0.01. To compare WT and SMA, unpaired t-test was used to determine statistical significance, and it was not significant. **g** Each dot represents a value from a neuron, WT untreated *n* = 204, WT CTRP3 treated *n* = 293, SMA untreated *n* = 200, SMA CTRP3 treated *n* = 200. Two way ANOVA with Sidak’s multiple comparison test was used to determine statistical significance; *p < 0.05, **p < 0.01, **h**-**i** Quantification of the ratio of the SUnSET signal in axons (per 20 μm) compared to average signal in soma. **h** Ratio between soma and axons were increased by CTRP3 treatment, WT *N* = 8, SMA *N* = 8. Each dot represents average data from one independent neuron culture. Therefore, data were only compared in the same culture with and without CTRP3 treatment. Two-tailed paired student’s t-test was used to determine statistical significance. ***p <  0.001 and **p < 0.01. To compare WT and SMA, unpaired t-test was used to determine statistical significance. *p < 0.05. **i** Ratio between soma and axons in individual neurons, each dot represents a value from a neuron, WT untreated *n* = 204, WT CTRP3 treated *n* = 293, SMA untreated *n* = 200, SMA CTRP3 treated *n* = 200. Two way ANOVA with Sidak’s multiple comparison test was used to determine statistical significance; *p < 0.05, **p < 0.01, ***p < 0.001

Finally, we explored the functional impact of CTRP3 in motor neuron. Initially, we hypothesized that intrinsic defects in SMA muscles affect the physiology of motor neuron via a secreted-molecule related retrograde communication. In fact, we found that SMA muscle cells secrete reduced levels of CTRP3, a protein known for its neurotrophic factor like functions. Therefore, we further investigated the effects of CTRP3 in motor neurons. First, as one of the best described phenotypes of SMA motor neurons is impaired axonal growth, we tested whether exogenous CTRP3 treatment can restore this phenotype at an early stage of development. We treated 5 μg/ml CTRP3 to 2DIV motor neurons, and the length of Tau-positive neurites was measured after 24 h. The majority of 3DIV motor neurons show one dominant Tau-positive neurite (Fig. 6a). We also used choline acetyl transferase as a marker for motor neurons (Fig. 6a). We observed that 24 h of 5 μg/ml CTRP3 treatment enhanced axonal outgrowth in WT and SMA motor neurons (Fig. 6b). Second, we tested whether CTRP3 can increase the local protein synthesis efficiency in the axonal compartment. Previously, it has been reported that SMA neurons show an impaired local translation rate in actively growing axons [1, 37]. Therefore, we tested whether exogenous CTRP3 can enhance local translation rate in growing axons. We labelled newly synthesized proteins in 3DIV motor neurons by incubating the cells with a low puromycin concentration (1 μM) for 30 min, visualized these proteins with anti-puromycin antibody, and measured the protein synthesis efficiency in the axonal compartment of WT and SMA motor neurons. To assure our experimental design, we used two negative controls. On the one hand, we treated cells with 50 μM anisomycin, a protein synthesis blocker, 30 min prior puromycin incubation (Fig. 6c). On the other hand, we stained untreated (without puromycin incubation) neurons with puromycin antibody to check background signals and could not detect any signals (Additional file 2: Figure S12). In addition, 30 min of anisomycin treatment successfully reduced the protein synthesis in neurons and this could be measured by SUnSET (Fig. 6d, e and Additional file 2: Figure S13). With this optimized protocol, we measured the intensity of SUnSET signals in a 20 μm length section in distal axons. We always measured the most distal axons, adjacent to the growth cones. As 3DIV motor neurons are usually 120–150 μm long (Fig. 6b), the data obtained from the axonal area was usually 80–100 μm apart from the neuronal soma. This was to avoid the signals from proteins anterogradely trafficked from soma to axon. This experiment revealed that CTRP3 enhances protein synthesis in axonal compartment (Fig. 6d, f and g) and that SUnSET signal is more than 10-fold higher in soma compared to axon (Fig. 6h and i). Furthermore, while we cannot fully explain the mechanism at the moment, we found that the ratio of protein synthesis between soma and axons is reduced in SMA motor neurons compared to WT ones, and that this is enhanced by CTRP3 treatment (Fig. 6 h and i). As protein synthesis efficiency in soma is already low in SMA neurons, these results suggest that protein synthesis is even more impaired in SMA axons. Taken together, our findings imply that there are additional mechanisms regulating protein synthesis in axons, and that this mechanism is dysregulated in SMA and can be restored by CTRP3 treatment.

## Discussion

The present study provides the first line of evidence that muscles regulate neuronal protein synthesis via secretory pathway, and that this mechanism is dysregulated in a neuromuscular disease, spinal muscular atrophy. While it has been debated whether axonal local protein synthesis can take place in vivo or fully mature neurons, growing evidence supports that proteins can be synthesized in axons and that protein synthesis rate can be regulated by external signals such as neurotrophic factors and axon guidance molecules (recently reviewed in [13]). However, it remained elusive how protein synthesis is regulated at the neuromuscular junctions, the synapses between motor neuronal axons and muscle fibers. In this study, we report that muscles can regulate axonal local translation via a secreted molecule, CTRP3, and that this mechanism is mTOR dependent. Impaired axonal local protein synthesis has been observed in another neuromuscular disease, ALS [44]. As lower motor neurons have extremely long axons, we can postulate that sufficient axonal protein synthesis is particularly important for proper function of lower motor neurons, and that their dysfunction can cause motor neuron diseases such as ALS and SMA. However, the molecular mechanisms regulating axonal protein synthesis in lower motor neurons needs further investigation.

Most importantly, we found that CTRP3 increases the protein levels of SMN and VEGF in motor neurons. Needless to say, SMN is the disease-causing protein in SMA and the level of SMN protein highly correlates with severity and progress of the disease. In severe cases of type I SMA patients and severe SMA mice, defects in the vascular system are evident and it might be causative for the distal necrosis [2, 30]. Therefore, we can speculate that restoring CTRP3 levels in SMA would increase the levels of SMN and VEGF, and thereby attenuate SMA disease progression.

Muscle has been recognized as an important endocrine organ as it secretes various signaling molecules in response to physical activity. For example, it is known that muscle contraction elevates the secretion of growth factors such as IGF-1 and FGF-2, which stimulate bone formation [26]. In other context, muscle can release cytokines such as IL-6, IL-8 and IL-15 in response to physical activity [56]. Until now, as muscles and bone are physically directly connected, research of the muscle secretome has been focused on the function in bone physiology. Here, we provide the first evidence showing the influence of muscle on physiology of neurons.

Low serum CTRP3 levels are linked to metabolic disorders such as type 2 diabetic mellitus and obesity [46, 77]. Interestingly, SMA patients and mouse models show dysfunctional glucose metabolism and defects in the pancreas [6, 16]. Based on these findings we can speculate that reduced serum CTRP3 levels can be due to metabolic dysfunction observed in SMA. However, it is important to note that our initial screening was performed in vitro cell culture system, which is influenced by neither other organs nor glucose supply. Therefore, this data led us to conclude that reduced secretion of CTRP3 is rather due to intrinsic defects in SMN deficient muscle cells. While the role of CTRP3 has been actively investigated in liver and kidney [48, 57] and genetically modified CTRP3 levels in mouse models show obvious effects on liver function [69, 76], the role of CTRP3 in neuromuscular systems has not been investigated so far. It will be important to check the neuromuscular system in genetically modified CTRP3 mouse models. Taken together, our data strongly implies molecular interaction between the neuromuscular system and glucose metabolism at the organismal level.

It is hitherto unknown how CTRP3 works on intracellular signal transduction pathways in neurons. It has been reported that CTRP3 binds to the lysosomal proteins, LAMP1 and LIMP II in a hepatoma cell line [42]. While LIMP II is located in the cell membrane, LAMP1 is not much located on the cell surface [65]. At the moment, the nature of CTRP3 action in neuronal cell signaling remains unclear. However, these data implies that CTRP3 may be taken up by cells via endocytosis and processed by endo-lysosomal pathway similar to BDNF and adiponectins [10, 19]. As the endocytosis pathway is impaired in SMA cells [29, 32, 60], this hypothesis could explain why the effect of CTRP3 on mTOR activation was slower in SMA motor neurons than in WT ones (Fig. 5). Furthermore, SMN protein levels were highest after 6 h treatment in WT motor neurons, but they were increased till 24 h after treatment in SMA ones (Fig. 4). In addition to that, receptor mediated signal transduction pathways are often regulated by a clathrin-dependent endocytosis pathway [19], which is impaired in SMA [60]. Together, these data strongly suggest that SMA motor neurons show altered kinetics in CTRP3 down-stream pathways. The molecular mechanism of CTRP3 signal transduction needs to be further characterized.

While our presented work here is focused on one protein CTRP3, there are other interesting candidate proteins whose secretion is altered in SMA muscle cells. Among them, one good example is VAPA (vesicle-associated membrane protein, associated protein A). Interestingly, mutations in the VAPA homolog VAPB (vesicle-associated membrane protein, associated protein B) have been reported as causative mutations in amyotrophic lateral sclerosis and late-onset spinal muscular atrophy [52]. VAPA and VAPB can form heterodimers and play an important role in vesicle trafficking together with VAMP-1 and -2 at the synaptic site [74]. As vesicular trafficking is impaired in SMA [17, 29] and VAPA secretion is elevated in SMA muscles, the role of VAPA in SMA can be interesting for investigation. Further interesting candidates are collagens as COL4A1, COL4A2 and COL5A2 were differentially secreted by SMA muscles (Table 1). Collagens are a major component of the extracellular matrix- essential for intercellular connections and signal transduction- and required for the structural and functional integrity of the NMJ [12]. Interestingly, mutations in *Col4a1* and *Col4a2* can cause neuromuscular dysfunction, vascular defects, myopathies and neurological disorders including epilepsy and cortical malformations [11, 33, 38, 80]. As both SMA and Col4a1-related disorders clearly share a common feature, neuromuscular dysfunction and vascular abnormality, collagens are a highly interesting topic for future studies to identify a new neuro-pathogenic pathway.

## Conclusion

Here we present a novel mechanism regulating neuronal local protein synthesis. We found that secretion of CTRP3 is reduced in SMA muscle cell lines, its expression is reduced in vulnerable tibialis muscle tissues, and blood plasma of SMA mice. Furthermore, CTRP3 regulates global/axonal protein synthesis and axonal growth in motor neurons. Importantly, CTRP3 enhances protein synthesis of SMN via activating mTOR pathway. Taken together, this is the first report showing that muscle regulates neuronal protein synthesis via the secretory pathway and that this process is dysregulated in the genetic neuromuscular disease, SMA. These findings revealed a new layer of neuro- pathogenic mechanism underlying SMA and further suggest muscle as a therapeutic target tissue in neuromuscular diseases.

## Supplementary information

**Additional file 1: Table S1.** Primer sequences. **Table S2.** Antibodies and conditions for Western blot (WB) and immunofluorescence (IF). **Table S3.** Pathway analysis: Secretomes from H-2K^b^-BL6 muscle cells (top 20 pathways). **Table S4.** Pathway analysis: Differentially secreted proteins from control and *Smn* KD H-2K^b^-BL6 muscle cells (*p* < 0.05 and FDR < 0.05). **Table S5.** List of differentially expressed proteins in control and *Smn* KD H-2K^b^-BL6 muscle cells (*p* < 0.05). **Table S6.** Pathway analysis: differentially expressed proteins in control and *Smn* KD H-2K^b^-BL6 muscle cells (proteins *p* < 0.05 and pathways *p* < 0.05). **Table S7.** List of proteins regulated by CTRP3 treatment (NSC-34 cells, *p* < 0.05, fold change > 25%). **Table S8.** Pathway analysis: proteins regulated by CTRP3 treatment (NSC-34 cells, proteins with *p* < 0.1 and pathways with *p* < 0.05). (DOCX 43 kb)

**Additional file 2: Figure S1.** Optimization of AHA treatment. **Figure S2.** Smn KD muscle cell proteome. **Figure S3.** Muscle CTRP3 images: high resolution. **Figure S4.** CTRP3 in liver. **Figure S5.** CTRP3 in brain and spinal cord. **Figure S6.** Optimization of CTRP3 treatment in NSC-34 cells. **Figure S7.** qRT-PCR after CTRP3 treatment in motor neurons. **Figure S8.** CTRP3 does not alter phosphorylation of AKT (S473). **Figure S9.** CTRP3 enhances cap-dependent translation. **Figure S10.** A bar graph summerises mTOR activity after CTRP3 and/or WYE-687 dihydrochloride treatment. **Figure S11.** ERK pathway does not inhibit CTRP3-mediated elevation of SMN proteins. **Figure S12.** Images of puromycin NOT-treated neurons: negative control. **Figure S13.** Images of representative neurons (ChAT and TAU), related to Fig. 6. (PPTX 21380 kb)

**Additional file 3.** Data 1.

**Additional file 4.** Data 2.

## Data Availability

All data generated or analyzed during this study are included in this published article and its supplementary information files.
