# Supplementary material for: Muscle regulates mTOR dependent axonal local translation in motor neurons via CTRP3 secretion: implications for a neuromuscular disorder, spinal muscular atrophy
Source: Acta Neuropathol Commun. 2019 Oct 15;7:154. doi: 10.1186/s40478-019-0806-3 (PMC6794869; doi:10.1186/s40478-019-0806-3)
Supplement: Supplementary file 1 — Additional file 1: Table S1. Primer sequences. Table S2. Antibodies and conditions for Western blot (WB) and immunofluorescence (IF). Table S3. Pathway analysis: Secretomes from H-2Kb-BL6 muscle cells (top 20 pathways). Table S4. Pathway analysis: Differentially secreted proteins from control and Smn KD H-2Kb-BL6 muscle cells (p < 0.05 and FDR < 0.05). Table S5. List of differentially expressed proteins in control and Smn KD H-2Kb-BL6 muscle cells (p < 0.05). Table S6. Pathway analysis: differentially expressed proteins in control and Smn KD H-2Kb-BL6 muscle cells (proteins p < 0.05 and pathways p < 0.05). Table S7. List of proteins regulated by CTRP3 treatment (NSC-34 cells, p < 0.05, fold change > 25%). Table S8. Pathway analysis: proteins regulated by CTRP3 treatment (NSC-34 cells, proteins with p < 0.1 and pathways with p < 0.05). (DOCX 43 kb) [file 40478_2019_806_MOESM1_ESM.docx]

**Table S1:** Primer sequences

| **Applications** | **Name** | **Sequence** | **Expected size** | **Annealing Temperature (ºC)** |
| --- | --- | --- | --- | --- |
| qRT-PCR | Actb-fw | AGCCATGTACGTAGCCATCC | 201 | 60 |
|  | Actb-rev | CTCTCAGCTGTGGTGGTGAA |  | 60 |
|  | Ctrp3-fw | CAGCAATCAGAACAGTGGCA | 289 | 65 |
|  | Ctrp3-rev | GTTGCCCATTCTTAGCCAGACT |  | 65 |
|  | Smn-fw | ACTCCTCCAGATCGCTCAGA | 227 | 58 |
|  | Smn-rev | AGGGGGTGGCGGGATTATTG |  | 58 |
|  | SMN2-fw | ACTCCTCCAGATCGCTCAGA | 227 | 58 |
|  | SMN2-rev | AGGGGGTGGAGGAATTATTG |  | 58 |
|  | Vegf-fw | CGACAGAAGGAGAGCAGAAGTCCC | 256 | 65 |
|  | Vegf-rev | TGGCTTTGGTGAGGTTTGATCCGC |  | 65 |

VEGF primers: Müller SM, et al., PNAS, 2005

**Table S2:** Antibodies and conditions for Western blot (WB) and immunofluorescence (IF)

| **Antibodies** | **Host species/dilution** | **Manufacturer/ #catalog** |
| --- | --- | --- |
| anti-ACTB, HRP-conjugated | mouse; WB 1:10,000 | Proteintech; HRP-60008 |
| anti-AKT | rabbit; WB 1:2000 | Cell Signaling; #9272 |
| anti-pAKT (S473) | rabbit; WB 1:1000 | Cell Signaling; #9271 |
| BTX, AlexaFluor-555 | IF 1:700-1000 | Thermo Fisher Scientific; B35451 |
| anti-ChAT | rabbit; IF 1:100 | Thermo Fisher Scientific; PA5-26597 |
| anti-CTRP3 | goat; WB 1:2000 | R&D Systems; AF2436 |
| anti-CTRP3 | sheep; WB 1:300 | R&D Systems; AF7925 |
| anti-CTRP3 | rabbit; IF 1:500 | Thermo Fisher Scientific; PA-20149 |
| anti-ERK2 | rabbit; WB 1:1000 | Sigma-Aldrich; M7431 |
| anti-pERK1/2 (Thr202/Tyr204) | rabbit; WB 1:500 | Cell Signaling; #9101 |
| anti-GAPDH, HRP-conjugated | mouse; WB 1:5000 | Sigma-Aldrich; G9295 |
| anti-Laminin 2 alpha | rat; IF 1:400 | Abcam; ab11576 |
| anti-MYOG | rabbit; WB 1:5000 | Abcam; ab124800 |
| anti-MHC | mouse; IF 1:50 | Hybridoma bank; MF-20 |
| anti-NF-L | rabbit; IF 1:500 | Cell Signaling; #2837 |
| anti-S6K | rabbit; WB 1:2000 | Cell Signaling; #2708 |
| anti-pS6K (Thr389) | rabbit; WB 1:750 | Cell Signaling; #9234 |
| anti-SMN | mouse; WB 1:3000 | BD Biosciences; 610646 |
| anti-Tau | mouse; IF 1:1000 | Santa Cruz; sc-390476 |
| anti-VEGFA | rabbit; WB 1:1000 | Abcam; ab46154 |
| anti-4E-BP1 | rabbit; WB 1:1000 | Cell Signaling; #9644 |
| anti-p4E-BP1 (Thr37/46) | rabbit; WB 1:1000 | Cell Signaling; #2855 |
| anti-PTEN | rabbit; WB 1:1000 | Cell Signaling; #9188 |
| anti-S6 | mouse; WB 1:1000 | Cell Signaling; #2317 |
| anti-pS6 (Ser235/236) | rabbit; WB 1:1000 | Cell Signaling; #2211 |
| anti-mouse IgG, HRP-conjugated | goat; WB 1:3000 | Dianova; 115-035-146 |
| anti-goat IgG, HRP-conjugated | rabbit; WB: 1:5000 | Merck Millipore; 401504 |
| anti-rabbit IgG, HRP-conjugated | goat; WB 1:2000 | Cell Signaling; #7074 |
| anti-sheep IgG, HRP-conjugated | donkey; WB 1:5000 | Abcam; ab150177 |
| anti-mouse IgG, AlexaFluor488-conjugated | goat; IF 1:350 | Thermo Fisher Scientific; A11001 |
| anti-rabbit IgG, AlexaFluor488-conjugated | donkey; IF 1:350 | Thermo Fisher Scientific; A21206 |
| anti-rabbit IgG, AlexaFluor568-conjugated | donkey; IF 1:350 | Thermo Fisher Scientific; A10042 |
| anti-rat IgG, AlexaFluor568-conjugated | goat; IF 1:350 | Thermo Fisher Scientific; A11077 |

**Table S3.** Pathway analysis: Secretomes from H-2K^b^-BL6 muscle cells (top 20 pathways)

| **Pathway name (reactome)** | **P value** | **FDR** | **Entities found/total** |
| --- | --- | --- | --- |
| SCF-beta-TrCP mediated degradation of Emi1 | 1.11E-16 | 1.52E-14 | 41/55 |
| Autodegradation of Cdh1 by Cdh1:APC/C | 1.11E-16 | 1.52E-14 | 40/63 |
| Formation of a pool of free 40S subunits | 1.11E-16 | 1.52E-14 | 65/106 |
| GTP hydrolysis and joining of the 60S ribosomal subunit | 1.11E-16 | 1.52E-14 | 73/120 |
| L13a-mediated translational silencing of Ceruloplasmin expression | 1.11E-16 | 1.52E-14 | 72/120 |
| Nonsense Mediated Decay (NMD) independent of the Exon Junction Complex (EJC) | 1.11E-16 | 1.52E-14 | 60/101 |
| Eukaryotic Translation Termination | 1.11E-16 | 1.52E-14 | 57/106 |
| SRP-dependent cotranslational protein targeting to membrane | 1.11E-16 | 1.52E-14 | 61/119 |
| Neutrophil degranulation | 1.11E-16 | 1.52E-14 | 165/480 |
| Mitotic Anaphase | 1.11E-16 | 1.52E-14 | 80/208 |
| Separation of Sister Chromatids | 1.11E-16 | 1.52E-14 | 76/194 |
| Cross-presentation of soluble exogenous antigens (endosomes) | 1.11E-16 | 1.52E-14 | 40/53 |
| Vif-mediated degradation of APOBEC3G | 1.11E-16 | 1.52E-14 | 43/56 |
| Regulation of ornithine decarboxylase (ODC) | 1.11E-16 | 1.52E-14 | 39/52 |
| Regulation of expression of SLITs and ROBOs | 1.11E-16 | 1.52E-14 | 108/183 |
| CDT1 association with the CDC6:ORC:origin complex | 4.44E-16 | 5.68E-14 | 40/59 |
| Peptide chain elongation | 8.88E-16 | 1.07E-13 | 55/97 |
| Assembly of the pre-replicative complex | 1.11E-15 | 1.27E-13 | 46/68 |
| Degradation of AXIN | 1.89E-15 | 2.04E-13 | 40/57 |
| Regulation of activated PAK-2p34 by proteasome mediated degradation | 2.44E-15 | 2.39E-13 | 41/50 |

**Table S4.** Pathway analysis: Differentially secreted proteins from control and *Smn* KD H-2K^b^-BL6 muscle cells (p<0.05 and FDR <0.05)

| **Pathway name (reactome)** | **P value** | **FDR** | **Entities found/total** | **Protein names** |
| --- | --- | --- | --- | --- |
| Post-translational protein phosphorylation | 3.48E-06 | 0.001 | 5/109 | RCN1, SERPINC1, TIMP1, CP, LGALS1 |
| Collagen biosynthesis and modifying enzymes | 3.38E-05 | 0.007 | 4/76 | COL5A2, SERPINH1, COL4A1, COL4A2 |
| Collagen chain trimerization | 6.11E-05 | 0.007 | 3/44 | COL5A2, COL4A1, COL4A2 |
| Regulation of Insulin-like Growth Factor (IGF) transport and uptake by Insulin-like Growth Factor Binding Proteins (IGFBPs) | 6.61E-05 | 0.007 | 5/127 | RCN1, SERPINC1, TIMP1, CP, LGALS1 |
| Collagen formation | 1.01E-04 | 0.008 | 4/104 | COL5A2, SERPINH1, COL4A1, COL4A2 |
| Assembly of collagen fibrils and other multimeric structures | 2.81E-04 | 0.016 | 3/67 | COL5A2, COL4A1, COL4A2 |
| Collagen degradation | 2.92E-04 | 0.016 | 3/69 | COL5A2, COL4A1, COL4A2 |
| Anchoring fibril formation | 3.12E-04 | 0.016 | 2/15 | COL4A1;COL4A2 |
| NCAM1 interactions | 3.78E-04 | 0.016 | 3/44 | COL5A2, COL4A1, COL4A2 |
| Degradation of the extracellular matrix | 3.83E-04 | 0.016 | 4/148 | COL5A2, COL4A1, COL4A2, TIMP1 |
| Crosslinking of collagen fibrils | 7.92E-04 | 0.029 | 2/24 | COL4A1, COL4A2 |
| ECM proteoglycans | 0.001 | 0.035 | 3/79 | COL5A2, COL4A1, COL4A2 |
| Laminin interactions | 0.001 | 0.047 | 2/31 | COL4A1, COL4A2 |

**Table S5.** List of differentially expressed proteins in control and *Smn* KD H-2K^b^-BL6 muscle cells (p<0.05)

| **Gene name** | **Protein name** | **# of unique peptides** | **P value** | **Fold change**  ***Smn*KD/Con** | **GO/KEGG** |
| --- | --- | --- | --- | --- | --- |
| *Larp4b* | La ribonucleoprotein domain family, member 4B | 4 | 0.0411 | 1.588 | RNA binding |
| *NS1BP* | Influenza virus NS1A binding protein | 7 | 0.0014 | 1.318 | RNA splicing, actin cytoskeleton |
| *Syne1* | Spectrin repeat containing, nuclear envelope 1, Nesprin1 | 3 | 0.0129 | 1.315 | Receptor mediated endocytosis, muscle differentiation |
| *Tmem9b* | Transmembrane protein 9B | 2 | 0.0297 | 1.274 |  |
| *Gamt* | Guanidinoacetate N-methyltransferase | 5 | 0.0368 | 1.245 | Amine biosynthetic process, muscle contraction |
| *Lox* | Lysyl oxidase | 5 | 0.0201 | 1.203 | Blood vessel development, muscle cell homeostasis and fiber differentiation |
| *Ppib* | Peptidylprolyl isomerase B | 9 | 0.0032 | 1.195 |  |
| *Akr1b7* | Aldo-keto reductase family 1, member B7 | 1 | 0.0273 | 1.195 | Lipid and glucose metabolism |
| *Man2c1* | Alpha-mannosidase 2C1 | 6 | 0.0257 | 1.195 | Mannose metabolic process |
| *Idi1* | Isopentenyl-diphosphate delta isomerase | 6 | 0.0208 | 1.174 | Isopentenyl diphosphate biosynthetic process |
| *Smap1* | Small ArfGAP 1 | 2 | 0.0046 | 1.167 | Clathrin dependent endocytosis |
| *Bcat2* | Branched chain aminotransferase 2, mitochondrial | 6 | 0.0411 | 1.147 | Amine biosynthetic process |
| *Fip1* | Factor interacting with poly(A) polymerase 1 | 2 | 0.0445 | 1.143 | mRNA polyadenylation |
| *Myl1* | Myosin light chain 1 | 10 | 0.0432 | 1.125 | Muscle contraction, Ca^2+^ binding |
| *Rab2a* | RAB2A, member RAS oncogene family | 8 | 0.0274 | 1.113 | AMPK pathway, vesicle mediated transport |
| *Rrm1* | Ribonucleotide reductase M1 | 14 | 0.0295 | 1.104 | ATP binding, terpenoid backbone biosynthesis |
| *Nasp* | Nuclear autoantigenic sperm protein | 6 | 0.0035 | 1.100 | Cell cycle, proliferation |
| *Prpf4b* | Pre-mRNA processing factor 4B | 4 | 0.0284 | 1.085 | mRNA processing, spliceosome |
| *Plaa* | Phospholipase A-2-activating protein | 25 | 0.0139 | 1.062 | Macroautophagy, synaptic vesicle recycling |
| *Limd1* | LIM domains containing 1 | 2 | 0.0408 | 0.967 | Cell junction, RISC complex |
| *Edc3* | Enhancer of mRNA decapping 3 | 2 | 0.0236 | 0.903 | Cytoplasmic mRNA processing body assembly, mRNA decay |
| *Snf5* (*Smarcb1*) | SWI/SNF-related matrix-associated actin-dependent regulator of chromatin subfamily B member 1 | 2 | 0.0344 | 0.884 | ATP-dependent chromatin-remodeling complex |
| Prkca | Protein kinase C alpha type | 2 | 0.0224 | 0.874 | VEGF, WNT signaling, smooth muscle contraction |
| *Gemin5* | Gem-associated protein 5 | 6 | 0.0242 | 0.868 | SMN complex, translation, spliceosomal snRNP assembly |
| *Ptk7* | Inactive tyrosine-protein kinase 7 | 12 | 0.0475 | 0.861 | Wnt signaling, angiogenesis |
| *Chkb* | Choline/ethanolamine kinase | 3 | 0.0150 | 0.857 | Glycerophospholipid metabolism |
| *Usp7* | Ubiquitin carboxyl-terminal hydrolase 7 | 15 | 0.0265 | 0.845 | Protein deubiquitination, protein stability |
| *Ppp1r3a* | Protein phosphatase 1 regulatory subunit 3A | 2 | 0.0484 | 0.840 | Glycogen metabolic process, serine/threonine phosphatase activity |
| *Gapvd1* | GTPase-activating protein and VPS9 domain-containing protein 1 | 13 | 0.0425 | 0.745 | Clathrin mediated endocytosis, Glut4 trafficking |
| *Pex5* | Peroxisomal biogenesis factor 5 | 6 | 0.0008 | 0.571 | Peroxisome |

**Table S6.** Pathway analysis: differentially expressed proteins in control and *Smn* KD H-2K^b^-BL6 muscle cells (proteins p<0.05 and pathways p<0.05)

| **Pathway name** | **P value** | **FDR** | **Entities found/total** | **Proteins** |
| --- | --- | --- | --- | --- |
| Protein ubiquitination | 0.0225 | 0.445 | 3/84 | PEX5, USP7 |
| Peroxisomal protein import | 0.0231 | 0.445 | 2/67 | PEX5 |
| Collagen formation | 0.0346 | 0.445 | 2/104 | LOX, PPIB |
| mRNA decay by 5' to 3' exoribonuclease | 0.0443 | 0.445 | 1/21 | EDC3 |
| Processing of Intronless Pre-mRNAs | 0.0462 | 0.445 | 1/19 | FIP1 |
| E3 ubiquitin ligases ubiquitinate target proteins | 0.0479 | 0.445 | 2/61 | PEX5 |
| Crosslinking of collagen fibrils | 0.0482 | 0.445 | 1/24 | LOX |

**Table S7.** List of proteins regulated by CTRP3 treatment (NSC-34 cells, p<0.05, fold change >25%)

| **Gene name** | **Protein name** | **# of unique peptides** | **p-value** | **Fold change**  **SMNKD/Cont** | **GO/KEGG** |
| --- | --- | --- | --- | --- | --- |
| *Cks2* | Cyclin-dependent kinases regulatory subunit 2 | 4 | 0.0098 | 1.570 | Proliferation, cell cycle |
| *Naa40* | N-alpha-acetyltransferase 40 | 3 | 0.0496 | 1.532 | Histone acetylation |
| *Gga3* | ADP-ribosylation factor-binding protein GGA3 | 8 | 0.0323 | 1.531 | Lysosome, vesicle mediated transport |
| *Tmed9* | Transmembrane emp24 domain-containing protein 9 | 6 | 0.0057 | 1.496 | Vesicle-mediated transport, ER-Golgi |
| *Daglb* | Sn1-specific diacylglycerol lipase beta | 5 | 0.0073 | 1.488 | Neurogenesis  Neurotransmitter biogenesis |
| *Slc16a3* | Monocarboxylate transporter 4 | 6 | 0.0101 | 1.474 | Pyruvate metabolism and TCA cycle |
| *Exoc3* | Exocyst complex component 3 | 9 | 0.0255 | 1.470 | Tight junction, cadherin and SNARE binding |
| *Stx6* | Syntaxin-6 | 3 | 0.0326 | 1.463 | SNARE interactions in vesicular transport, endocytic recycling |
| *Tbcel* | Tubulin-specific chaperone cofactor E-like protein | 7 | 0.0433 | 1.423 | Cytoskeleton |
| *Ptbp3* | Polypyrimidine tract-binding protein 3 | 5 | 0.0076 | 1.408 | RNA splicing |
| *Pbk* | Lymphokine-activated killer T-cell-originated protein kinase | 3 | 0.0298 | 1.386 | ATP binding, kinase |
| *Mettl26* | Methyltransferase-like 26 | 5 | 0.0437 | 1.377 |  |
| *Mrps31* | 28S ribosomal protein S31, mitochondrial | 13 | 0.0358 | 1.365 | Ribosome |
| *Pts* | 6-pyruvoyl tetrahydrobiopterin synthase | 7 | 0.0382 | 1.356 | Tetrahydrobiopterin (BH4) synthesis, mitochondria |
| *Manf* | Mesencephalic astrocyte-derived neurotrophic factor | 12 | 0.0232 | 1.334 | growth factor activity, dopaminergic neuron differentiation |
| *Gabpa* | GA-binding protein alpha chain | 6 | 0.0089 | 1.330 | Transcriptional activation of mitochondrial biogenesis |
| *Akr1e* | 1,5-anhydro-D-fructose reductase | 12 | 0.0239 | 1.310 | oxidation-reduction process |
| *Cks1b* | Cyclin-dependent kinases regulatory subunit 1 | 3 | 0.0272 | 1.304 | Proliferation, cell cycle |
| *Anp32e* | Acidic leucine-rich nuclear phosphoprotein 32 family member E | 6 | 0.0154 | 1.303 | regulation of apoptotic process, chromatin organization |
| *Bola2* | BolA-like protein 2 | 7 | 0.0137 | 1.286 |  |
| *Akt2* | RAC-beta serine/threonine-protein kinase | 4 | 0.0222 | 1.278 | VEGFA pathway, kinase activity |
| *Pgam1* | Phosphoglycerate mutase 1 | 17 | 0.0326 | 1.271 | Glucose metabolism |
| *Ubac1* | Ubiquitin-associated domain-containing protein 1 | 3 | 0.0037 | 1.260 | Ubiquitination |
| *Uqcrb* | Cytochrome b-c1 complex subunit 7 | 11 | 0.0447 | 1.258 | Mitochondria, Alzheimer’s disease |
| *Nudcd3* | NudC domain-containing protein 3 | 12 | 0.0362 | 1.257 | Cytoplasmic dynein complex, cilium assembly |
| *Mtpn* | Myotrophin | 10 | 0.0387 | 1.256 | Axon, cerebellar granule cell differentiation |
| *Fbxw8* | F-box/WD repeat-containing protein 8 | 13 | 0.0402 | 1.254 | ubiquitination |
| *Nol11* | Nucleolar protein 11 | 6 | 0.0498 | 0.747 | Ribosome biogenesis, rRNA processing |
| *Wasf1* | Wiskott-Aldrich syndrome protein family member 1 | 11 | 0.0471 | 0.739 | VEGF pathway, actin binding |
| *Arf5* | ADP-ribosylation factor 5 | 8 | 0.0341 | 0.704 | Vesicle-mediated transport, COPI mediated transport |
| *Dhcr24* | Delta(24)-sterol reductase | 6 | 0.0210 | 0.688 | Cholesterol biosynthesis |
| *Mtpap* | Poly(A) RNA polymerase, mitochondrial | 9 | 0.0002 | 0.678 | Histone mRNA process, mRNA polyadenylation |
| *Ghitm* | Growth hormone-inducible transmembrane protein | 4 | 0.0219 | 0.634 | Apoptotic process |

**Table S8.** Pathway analysis: proteins regulated by CTRP3 treatment (NSC-34 cells, proteins with p<0.1 and pathways with p<0.05)

| **Pathway name** | **P value** | **FDR** | **Entities found/total** | **Proteins** |
| --- | --- | --- | --- | --- |
| Mitochondrial translation initiation | 0.0044 | 0.829 | 8/96 | MRPL3, MRPL4, MRPL22, MRPS31, MRPS24, MRPL50, MTIF2, MRPL30 |
| Formation of a pool of free 40S subunits | 0.0046 | 0.829 | 7/106 | RPS26, RPL18A, EIF3I, RPS6, RPL13A, EIF3G, RPL19 |
| L13a-mediated translational silencing of Ceruloplasmin expression | 0.0069 | 0.829 | 8/120 | RPS26, RPL18A, EIF3I, RPS6, RPL13A, EIF3G, EIF2A, RPL19 |
| Glycolysis | 0.0072 | 0.829 | 8/110 | PKM, TPI1, PGK1, PGAM1, NUP88, HK2 |
| GTP hydrolysis and joining of the 60S ribosomal subunit | 0.0089 | 0.829 | 8/120 | RPS26, RPL18A, EIF3I, RPS6, RPL13A, EIF3G, EIF2A, RPL19 |
| Mitochondrial translation elongation | 0.0102 | 0.829 | 7/94 | MRPL3, MRPL4, MRPL22, MRPS31, MRPS24, MRPL50, MRPL30 |
| Mitochondrial translation termination | 0.0106 | 0.829 | 7/94 | MRPL3, MRPL4, MRPL22, MRPS31, MRPS24, MRPL50, MRPL30 |
| RNA Polymerase III Transcription Initiation From Type 2 Promoter | 0.0140 | 0.829 | 3/27 | GTF3C4, POLR3D, POLR3B |
| Formation of the ternary complex, and subsequently, the 43S complex | 0.0153 | 0.829 | 5/54 | RPS26, EIF3I, RPS6, EIF3G, EIF2A |
| RNA Polymerase III Transcription Initiation From Type 1 Promoter | 0.0154 | 0.829 | 3/28 | GTF3C4, POLR3D, POLR3B |
| TP53 Regulates Metabolic Genes | 0.0155 | 0.829 | 9/125 | PRDX2, COX6B1, COX6A1, YWHAE, YWHAB, AKT2, PTEN |
| Insulin receptor recycling | 0.0169 | 0.829 | 3/28 | ATP6V1A, ATP6V1E1 |
| Eukaryotic Translation Termination | 0.0176 | 0.829 | 6/106 | RPS26, RPL18A, RPS6, RPL13A, APEH, RPL19 |
| G2/M Checkpoints | 0.0197 | 0.829 | 9/154 | PSMA3, YWHAE, PSMC1, YWHAB, BABAM1, PSMB2, MCM6, MRE11A, MYT1 |
| Mitochondrial translation | 0.0200 | 0.829 | 8/102 | MRPL3, MRPL4, MRPL22, MRPS31, MRPS24, MRPL50, MTIF2, MRPL30 |
| Proton-coupled monocarboxylate transport | 0.0209 | 0.829 | 2/12 | SLC16A1, SLC16A3 |
| Glucose metabolism | 0.0224 | 0.829 | 9/141 | G6PC3, PKM, TPI1, PGK1, PGAM1, NUP88, HK2 |
| SRP-dependent cotranslational protein targeting to membrane | 0.0238 | 0.829 | 6/119 | RPS26, RPL18A, RPS6, RPL13A, SRP72, RPL19 |
| Translation initiation complex formation | 0.0264 | 0.829 | 5/62 | RPS26, EIF3I, RPS6, EIF3G, EIF2A |
| Activation of BAD and translocation to mitochondria | 0.0314 | 0.829 | 4/19 | PPP3R1, YWHAE, YWHAB, AKT2 |
| Utilization of Ketone bodies | 0.0316 | 0.829 | 2/14 | OXCT1 |
| Ribosomal scanning and start codon recognition | 0.0321 | 0.829 | 5/64 | RPS26, EIF3I, RPS6, EIF3G, EIF2A |
| Recruitment of NuMA to mitotic centrosomes | 0.0346 | 0.829 | 5/97 | YWHAE, DCTN1, CETN2, FGFR1OP, HSP90AA1 |
| Cap-dependent Translation Initiation | 0.0426 | 0.829 | 9/130 | RPS26, EIF2B2, RPL18A, EIF3I, RPS6, RPL13A, EIF3G, EIF2A, RPL19 |
| AURKA Activation by TPX2 | 0.0429 | 0.829 | 5/74 | YWHAE, DCTN1, CETN2, FGFR1OP, HSP90AA1 |
| Eukaryotic Translation Initiation | 0.0435 | 0.829 | 9/130 | RPS26, EIF2B2, RPL18A, EIF3I, RPS6, RPL13A, EIF3G, EIF2A, RPL19 |
| Regulation of RAS by GAPs | 0.0450 | 0.829 | 4/70 | PSMA3, NRAS, PSMC1, PSMB2 |
| Respiratory electron transport | 0.0471 | 0.829 | 8/115 | COX6B1, COX6A1, UQCRQ, UQCRFS1, UQCRB, NDUFV3, NDUFS3, NDUFC2 |
| Translation | 0.0471 | 0.829 | 19/339 | MRPL3, MRPL4, MRPL22, RPS26, EIF3I, RPL18A, MRPS31, RPS6, MRPS24, EIF3G, SRP72, EIF2A, APEH, RPL19, EIF2B2, RPL13A, MRPL50, MTIF2, MRPL30 |
