# Supplementary material for: Muscle regulates mTOR dependent axonal local translation in motor neurons via CTRP3 secretion: implications for a neuromuscular disorder, spinal muscular atrophy
Source: Acta Neuropathol Commun. 2019 Oct 15;7:154. doi: 10.1186/s40478-019-0806-3 (PMC6794869; doi:10.1186/s40478-019-0806-3)
Supplement: Supplementary file 2 — Additional file 2: Figure S1. Optimization of AHA treatment. Figure S2. Smn KD muscle cell proteome. Figure S3. Muscle CTRP3 images: high resolution. Figure S4. CTRP3 in liver. Figure S5. CTRP3 in brain and spinal cord. Figure S6. Optimization of CTRP3 treatment in NSC-34 cells. Figure S7. qRT-PCR after CTRP3 treatment in motor neurons. Figure S8. CTRP3 does not alter phosphorylation of AKT (S473). Figure S9. CTRP3 enhances cap-dependent translation. Figure S10. A bar graph summerises mTOR activity after CTRP3 and/or WYE-687 dihydrochloride treatment. Figure S11. ERK pathway does not inhibit CTRP3-mediated elevation of SMN proteins. Figure S12. Images of puromycin NOT-treated neurons: negative control. Figure S13. Images of representative neurons (ChAT and TAU), related to Fig. 6. (PPTX 21380 kb) [file 40478_2019_806_MOESM2_ESM.pptx]

## Slide 1
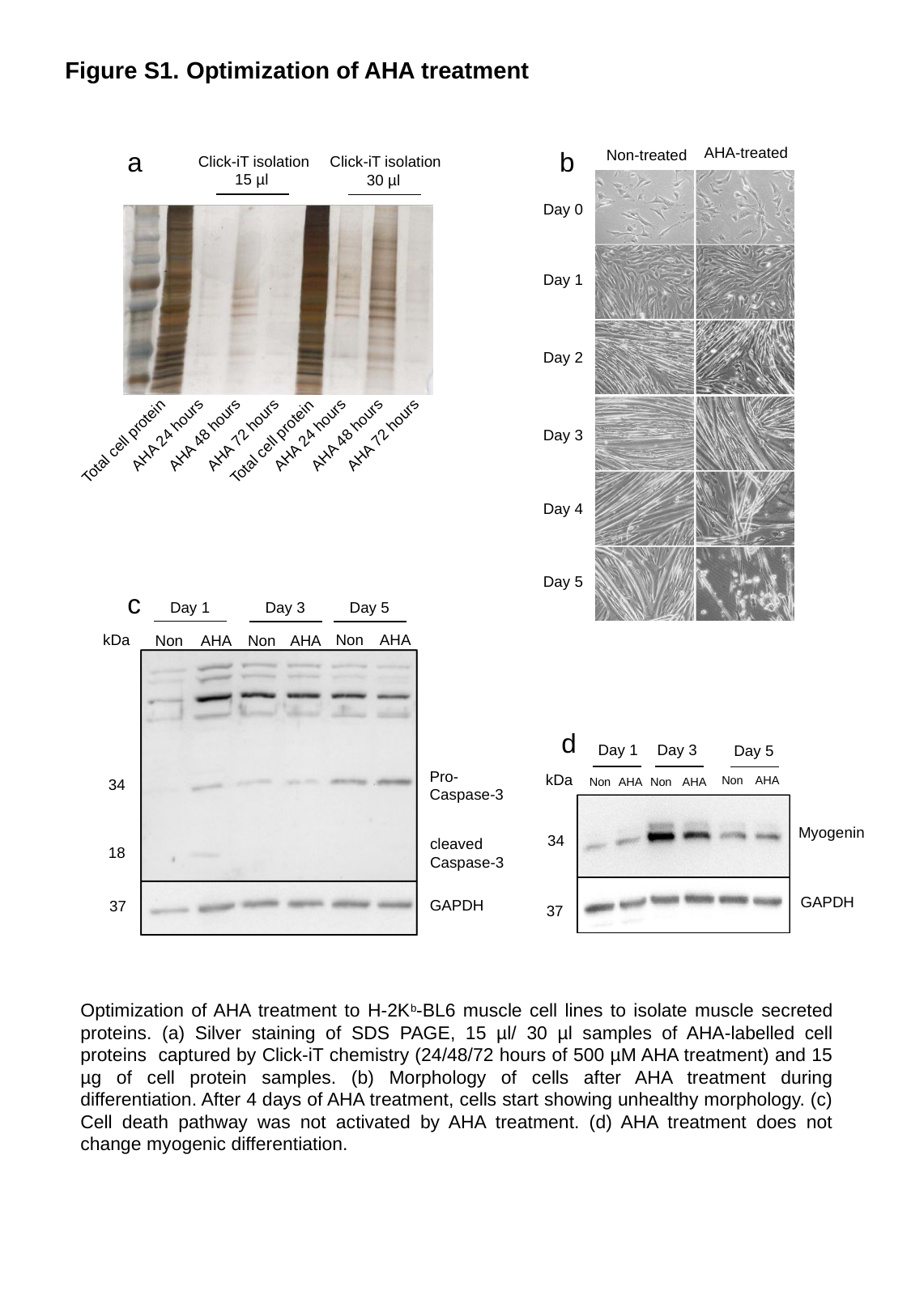

Figure S1. Optimization of AHA treatment
AHA-treated
a
b
Non-treated
Click-iT isolation
15 µl
Click-iT isolation
30 µl
Day 0
Day 1
Day 2
AHA 72 hours
AHA 72 hours
Day 3
AHA 24 hours
AHA 48 hours
AHA 24 hours
AHA 48 hours
Total cell protein
Total cell protein
Day 4
Day 5
c
Day 1
Day 3
Day 5
kDa
Non
AHA
Non
AHA
Non
AHA
d
Day 3
Day 1
Day 5
Pro-
Caspase-3
kDa
Non
AHA
AHA
AHA
Non
Non
34
Myogenin
34
cleaved
Caspase-3
18
GAPDH
GAPDH
37
37
Optimization of AHA treatment to H-2Kb-BL6 muscle cell lines to isolate muscle secreted proteins. (a) Silver staining of SDS PAGE, 15 µl/ 30 µl samples of AHA-labelled cell proteins captured by Click-iT chemistry (24/48/72 hours of 500 µM AHA treatment) and 15 µg of cell protein samples. (b) Morphology of cells after AHA treatment during differentiation. After 4 days of AHA treatment, cells start showing unhealthy morphology. (c) Cell death pathway was not activated by AHA treatment. (d) AHA treatment does not change myogenic differentiation.

## Slide 2
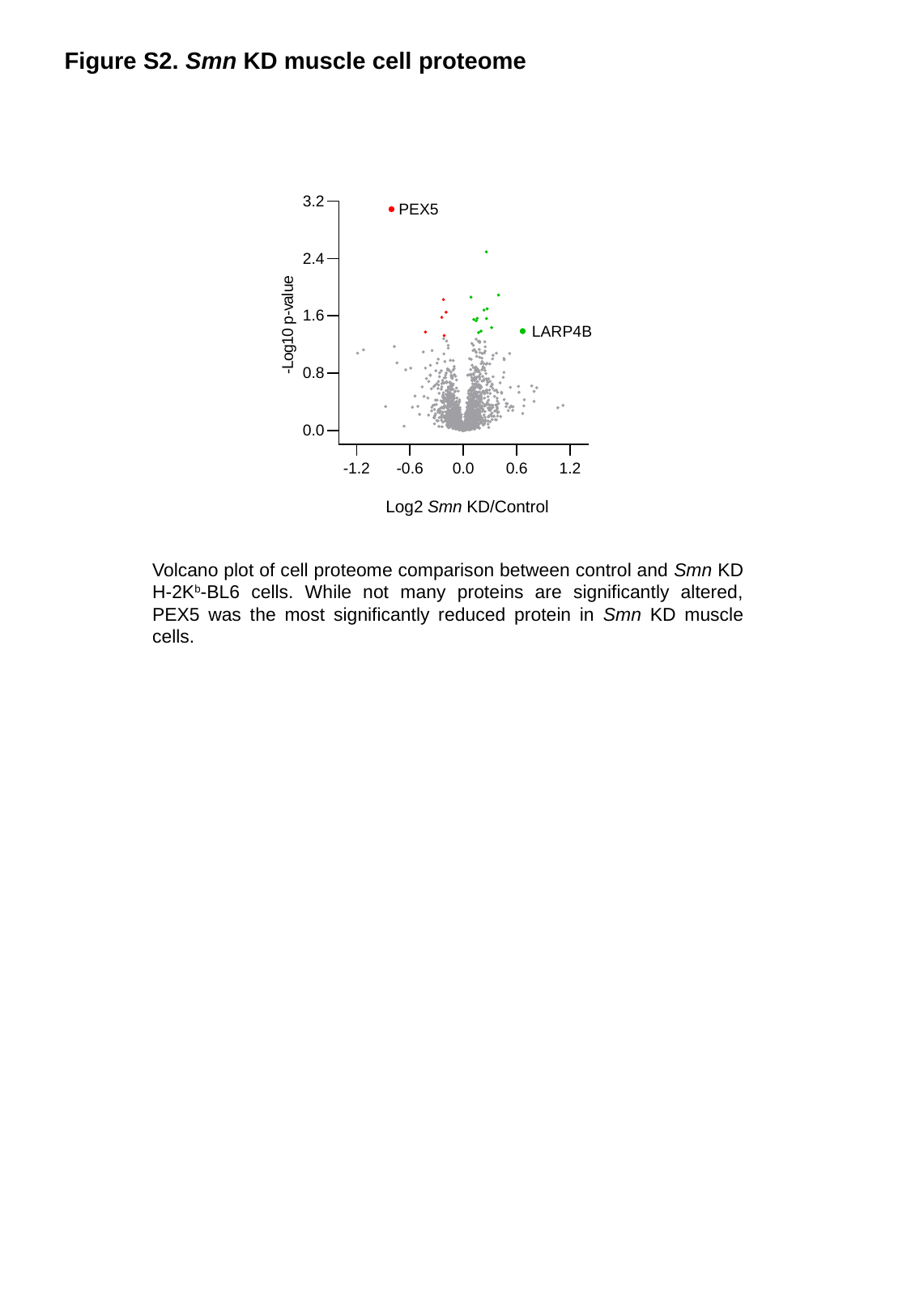

Figure S2. Smn KD muscle cell proteome
Log2 Smn KD/Control
Volcano plot of cell proteome comparison between control and Smn KD H-2Kb-BL6 cells. While not many proteins are significantly altered, PEX5 was the most significantly reduced protein in Smn KD muscle cells.

## Slide 3
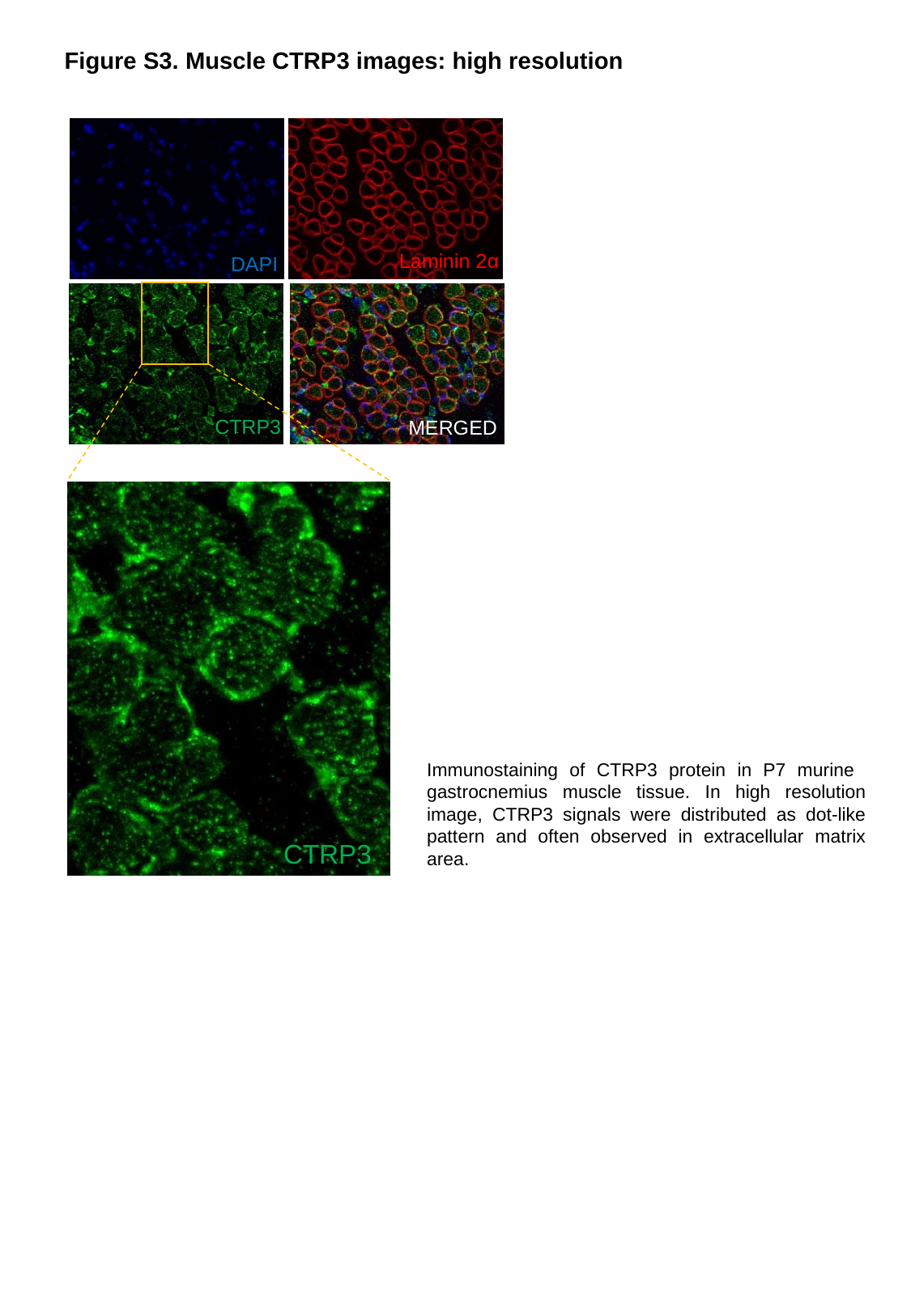

Figure S3. Muscle CTRP3 images: high resolution
Laminin 2α
DAPI
CTRP3
MERGED
Immunostaining of CTRP3 protein in P7 murine gastrocnemius muscle tissue. In high resolution image, CTRP3 signals were distributed as dot-like pattern and often observed in extracellular matrix area.
CTRP3

## Slide 4
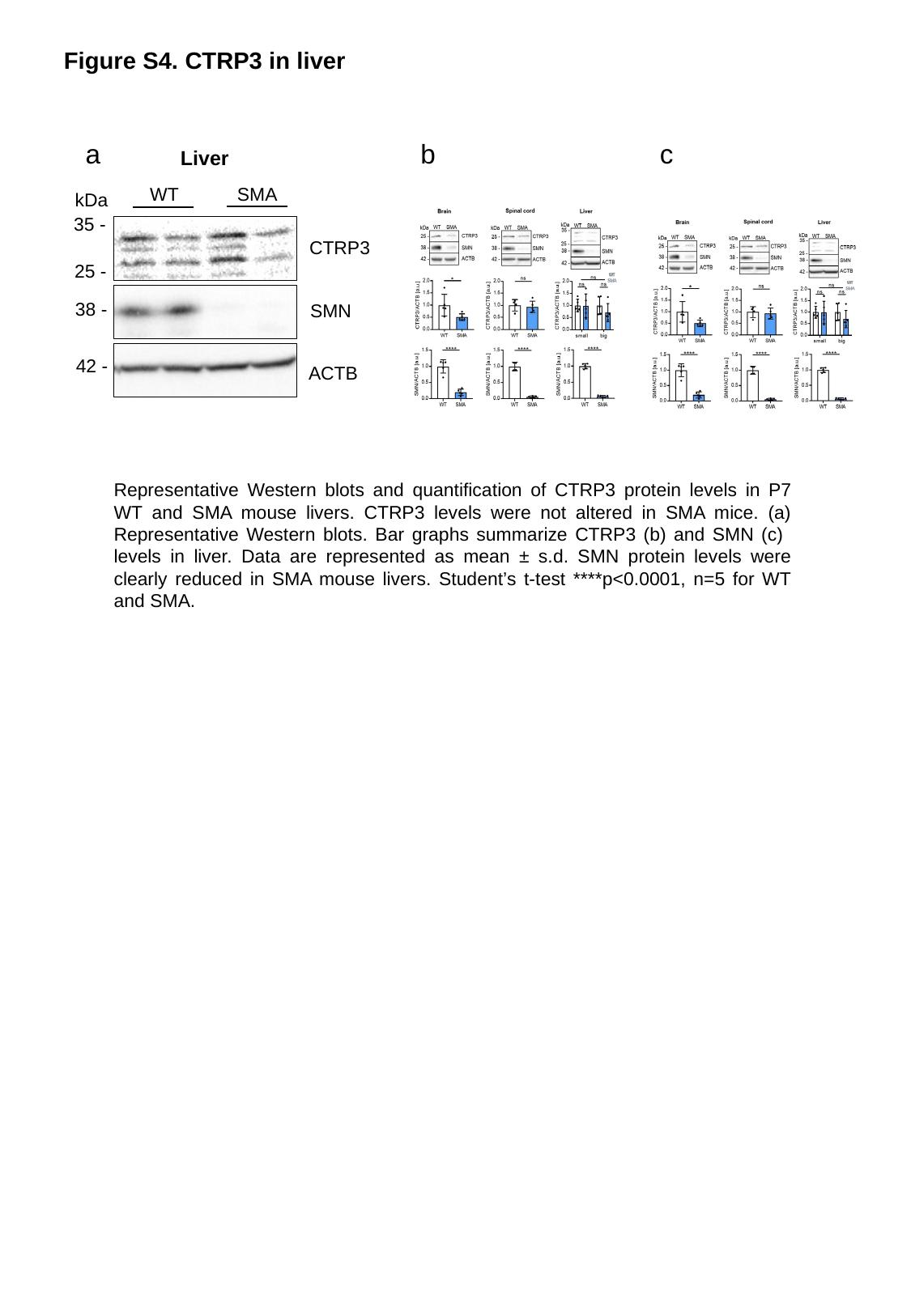

Figure S4. CTRP3 in liver
c
b
a
Liver
WT
SMA
kDa
35 -
CTRP3
25 -
38 -
SMN
42 -
ACTB
Representative Western blots and quantification of CTRP3 protein levels in P7 WT and SMA mouse livers. CTRP3 levels were not altered in SMA mice. (a) Representative Western blots. Bar graphs summarize CTRP3 (b) and SMN (c) levels in liver. Data are represented as mean ± s.d. SMN protein levels were clearly reduced in SMA mouse livers. Student’s t-test ****p<0.0001, n=5 for WT and SMA.

## Slide 5
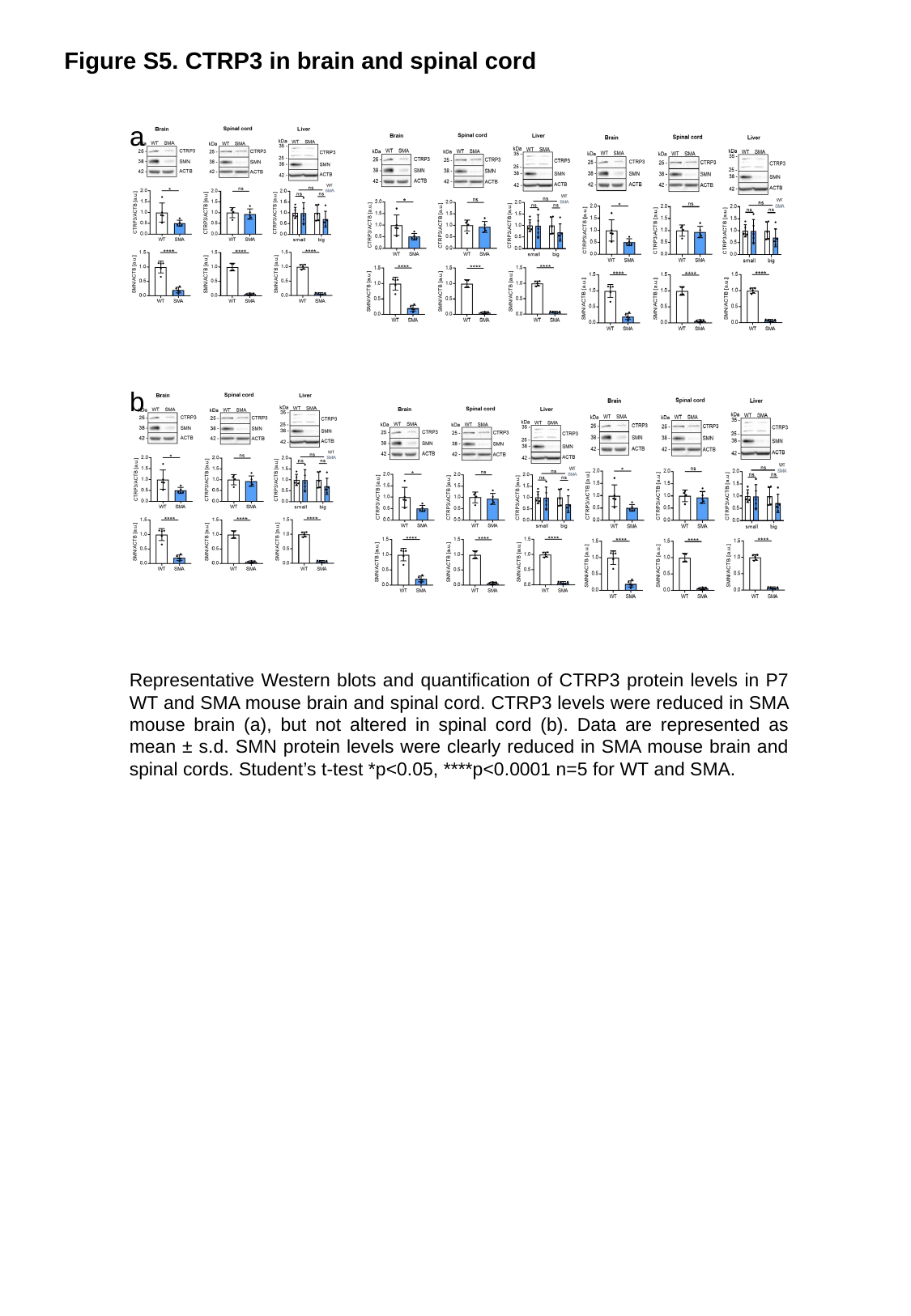

Figure S5. CTRP3 in brain and spinal cord
a
b
Representative Western blots and quantification of CTRP3 protein levels in P7 WT and SMA mouse brain and spinal cord. CTRP3 levels were reduced in SMA mouse brain (a), but not altered in spinal cord (b). Data are represented as mean ± s.d. SMN protein levels were clearly reduced in SMA mouse brain and spinal cords. Student’s t-test *p<0.05, ****p<0.0001 n=5 for WT and SMA.

## Slide 6
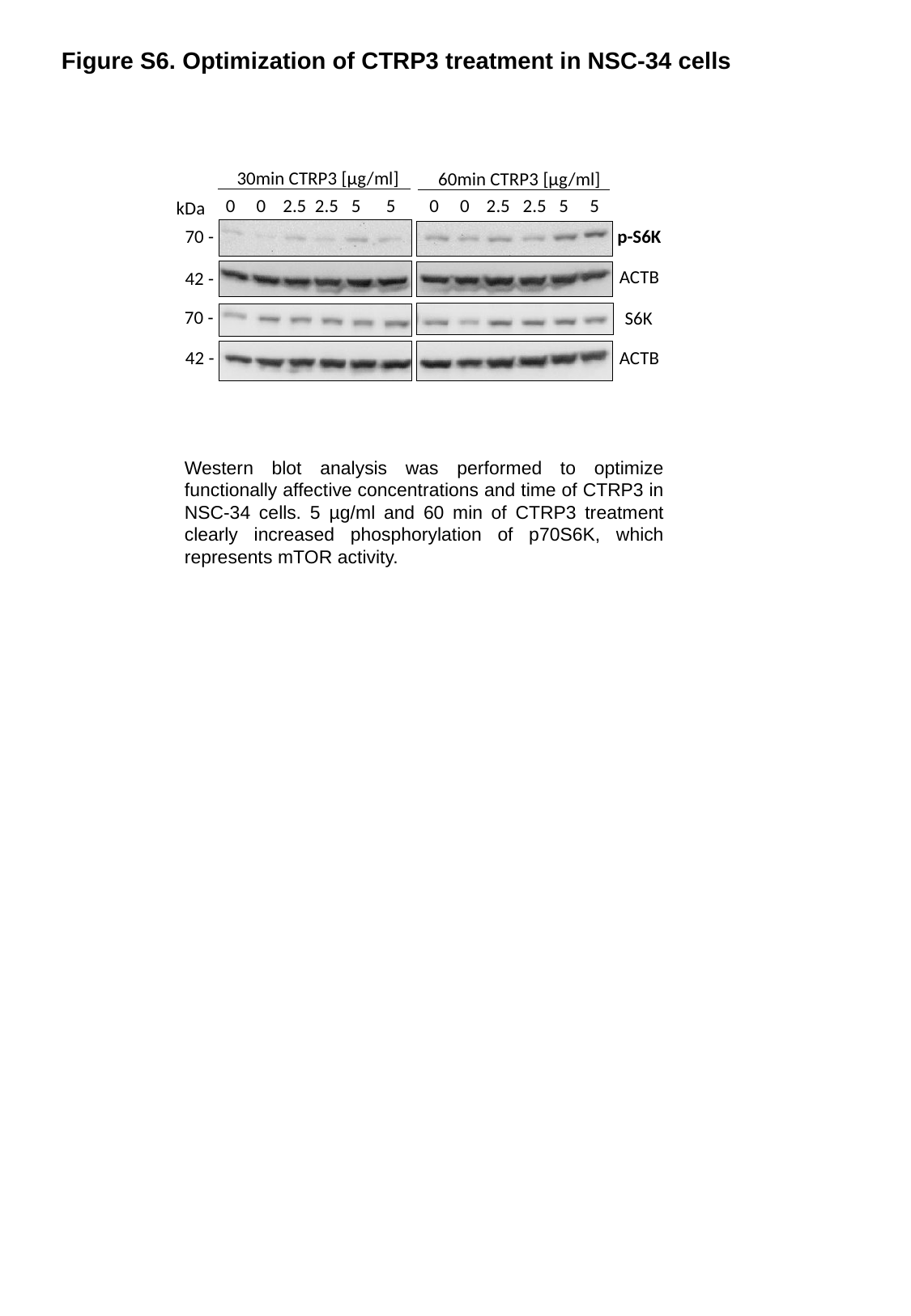

Figure S6. Optimization of CTRP3 treatment in NSC-34 cells
30min CTRP3 [µg/ml]
60min CTRP3 [µg/ml]
0 0 2.5 2.5 5 5
0 0 2.5 2.5 5 5
p-S6K
ACTB
S6K
ACTB
kDa
70 -
42 -
70 -
42 -
Western blot analysis was performed to optimize functionally affective concentrations and time of CTRP3 in NSC-34 cells. 5 µg/ml and 60 min of CTRP3 treatment clearly increased phosphorylation of p70S6K, which represents mTOR activity.

## Slide 7
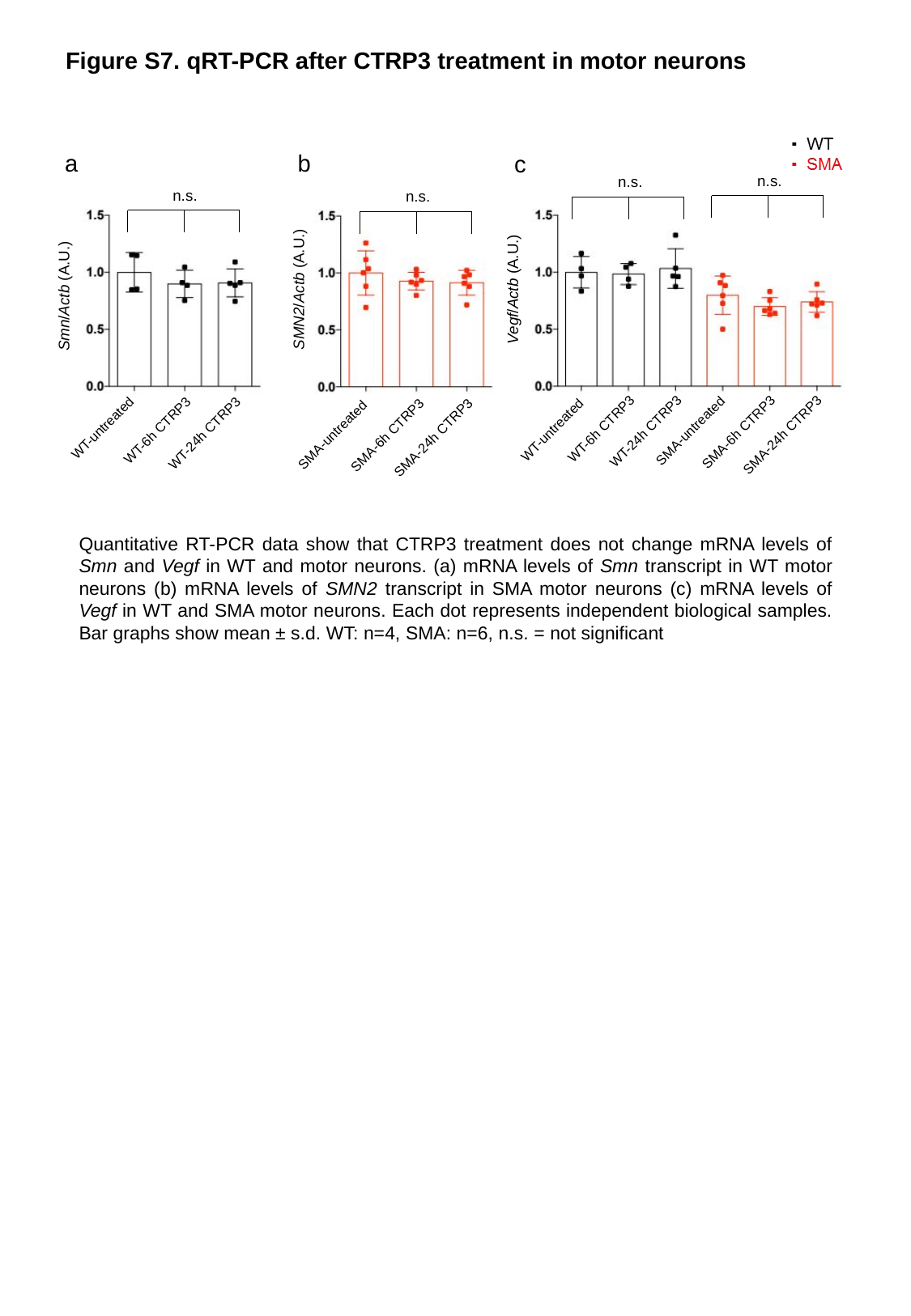

Figure S7. qRT-PCR after CTRP3 treatment in motor neurons
b
a
c
n.s.
n.s.
Vegf/Actb (A.U.)
WT-6h CTRP3
WT-untreated
SMA-untreated
WT-24h CTRP3
SMA-6h CTRP3
SMA-24h CTRP3
n.s.
Smn/Actb (A.U.)
WT-untreated
WT-6h CTRP3
WT-24h CTRP3
n.s.
SMN2/Actb (A.U.)
SMA-untreated
SMA-6h CTRP3
SMA-24h CTRP3
Quantitative RT-PCR data show that CTRP3 treatment does not change mRNA levels of Smn and Vegf in WT and motor neurons. (a) mRNA levels of Smn transcript in WT motor neurons (b) mRNA levels of SMN2 transcript in SMA motor neurons (c) mRNA levels of Vegf in WT and SMA motor neurons. Each dot represents independent biological samples. Bar graphs show mean ± s.d. WT: n=4, SMA: n=6, n.s. = not significant

## Slide 8
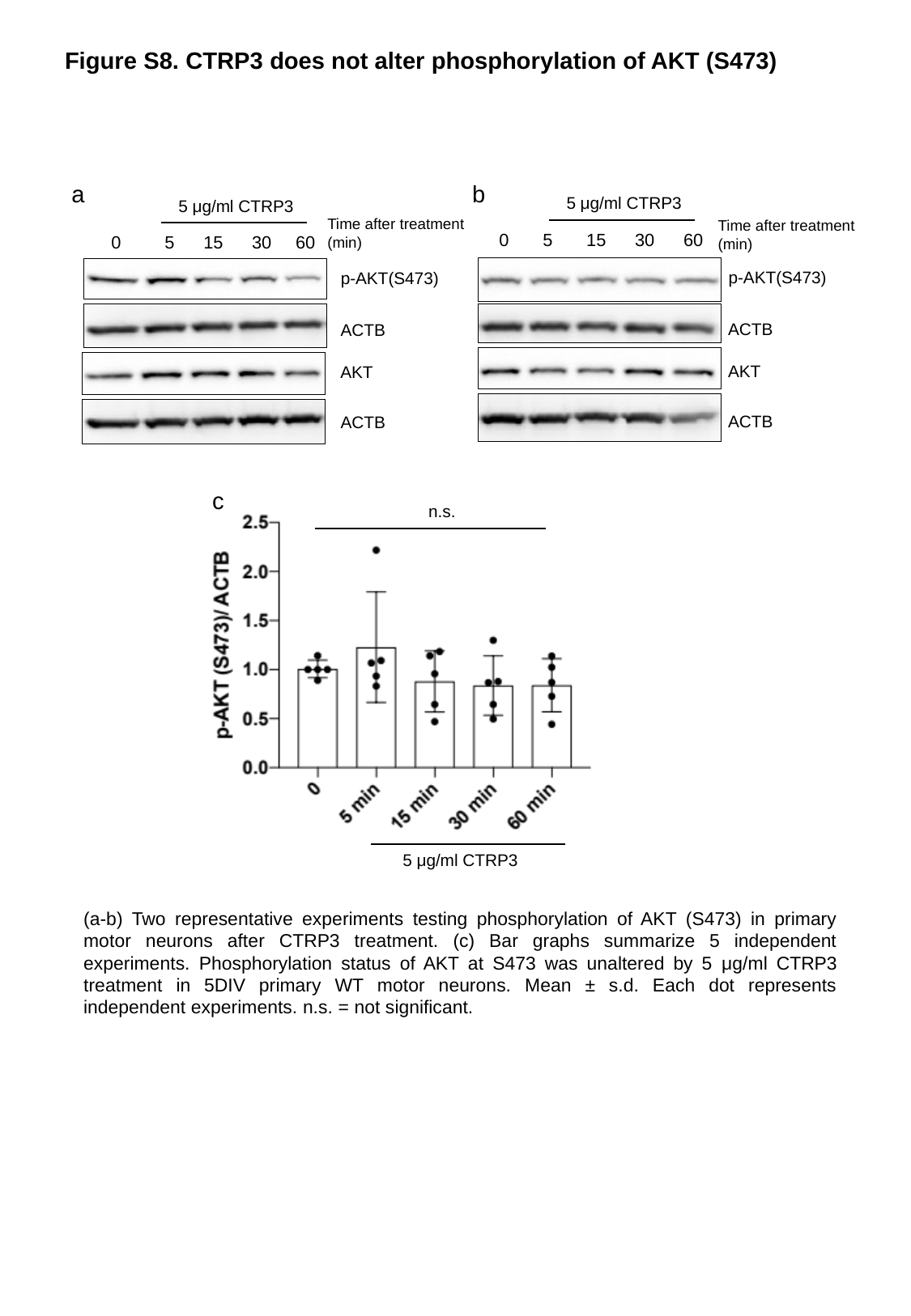

Figure S8. CTRP3 does not alter phosphorylation of AKT (S473)
a
b
5 μg/ml CTRP3
5 μg/ml CTRP3
Time after treatment
(min)
Time after treatment
(min)
0 5 15 30 60
0 5 15 30 60
p-AKT(S473)
p-AKT(S473)
ACTB
ACTB
AKT
AKT
ACTB
ACTB
c
n.s.
5 μg/ml CTRP3
(a-b) Two representative experiments testing phosphorylation of AKT (S473) in primary motor neurons after CTRP3 treatment. (c) Bar graphs summarize 5 independent experiments. Phosphorylation status of AKT at S473 was unaltered by 5 μg/ml CTRP3 treatment in 5DIV primary WT motor neurons. Mean ± s.d. Each dot represents independent experiments. n.s. = not significant.

## Slide 9
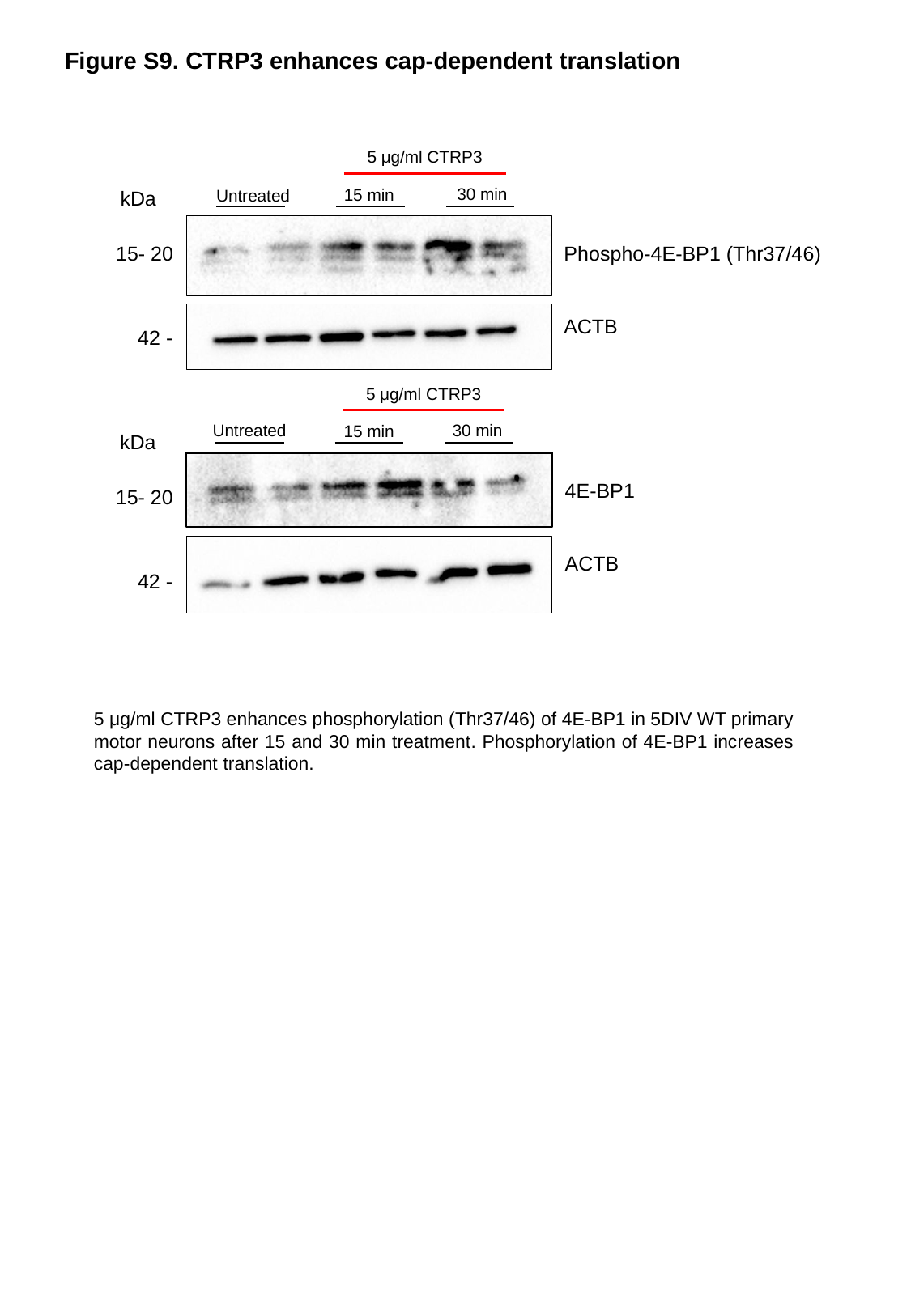

Figure S9. CTRP3 enhances cap-dependent translation
5 μg/ml CTRP3
30 min
15 min
Untreated
kDa
15- 20
Phospho-4E-BP1 (Thr37/46)
ACTB
42 -
5 μg/ml CTRP3
Untreated
30 min
15 min
kDa
4E-BP1
15- 20
ACTB
42 -
5 μg/ml CTRP3 enhances phosphorylation (Thr37/46) of 4E-BP1 in 5DIV WT primary motor neurons after 15 and 30 min treatment. Phosphorylation of 4E-BP1 increases cap-dependent translation.

## Slide 10
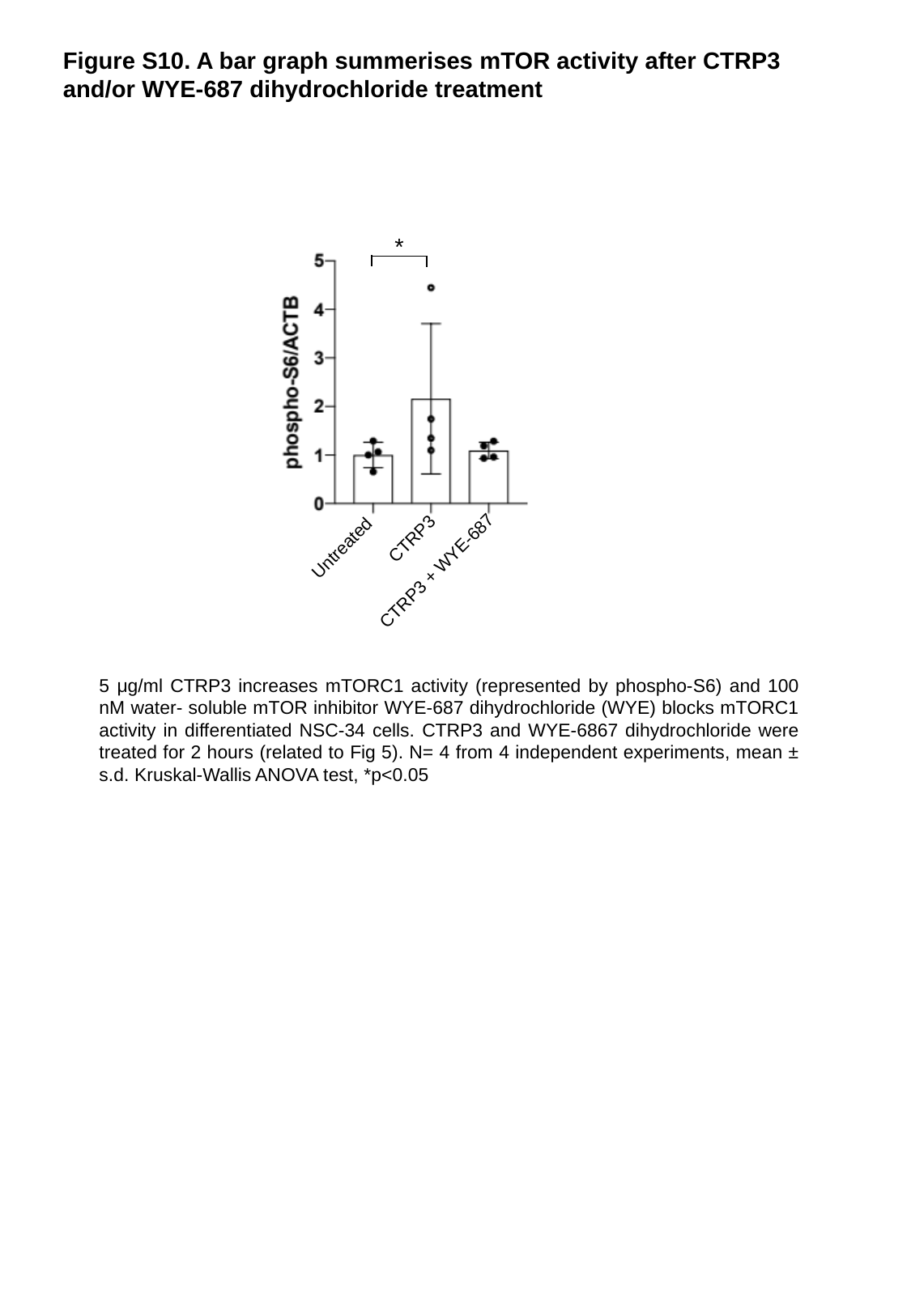

Figure S10. A bar graph summerises mTOR activity after CTRP3 and/or WYE-687 dihydrochloride treatment
*
CTRP3
Untreated
CTRP3 + WYE-687
5 μg/ml CTRP3 increases mTORC1 activity (represented by phospho-S6) and 100 nM water- soluble mTOR inhibitor WYE-687 dihydrochloride (WYE) blocks mTORC1 activity in differentiated NSC-34 cells. CTRP3 and WYE-6867 dihydrochloride were treated for 2 hours (related to Fig 5). N= 4 from 4 independent experiments, mean ± s.d. Kruskal-Wallis ANOVA test, *p<0.05

## Slide 11
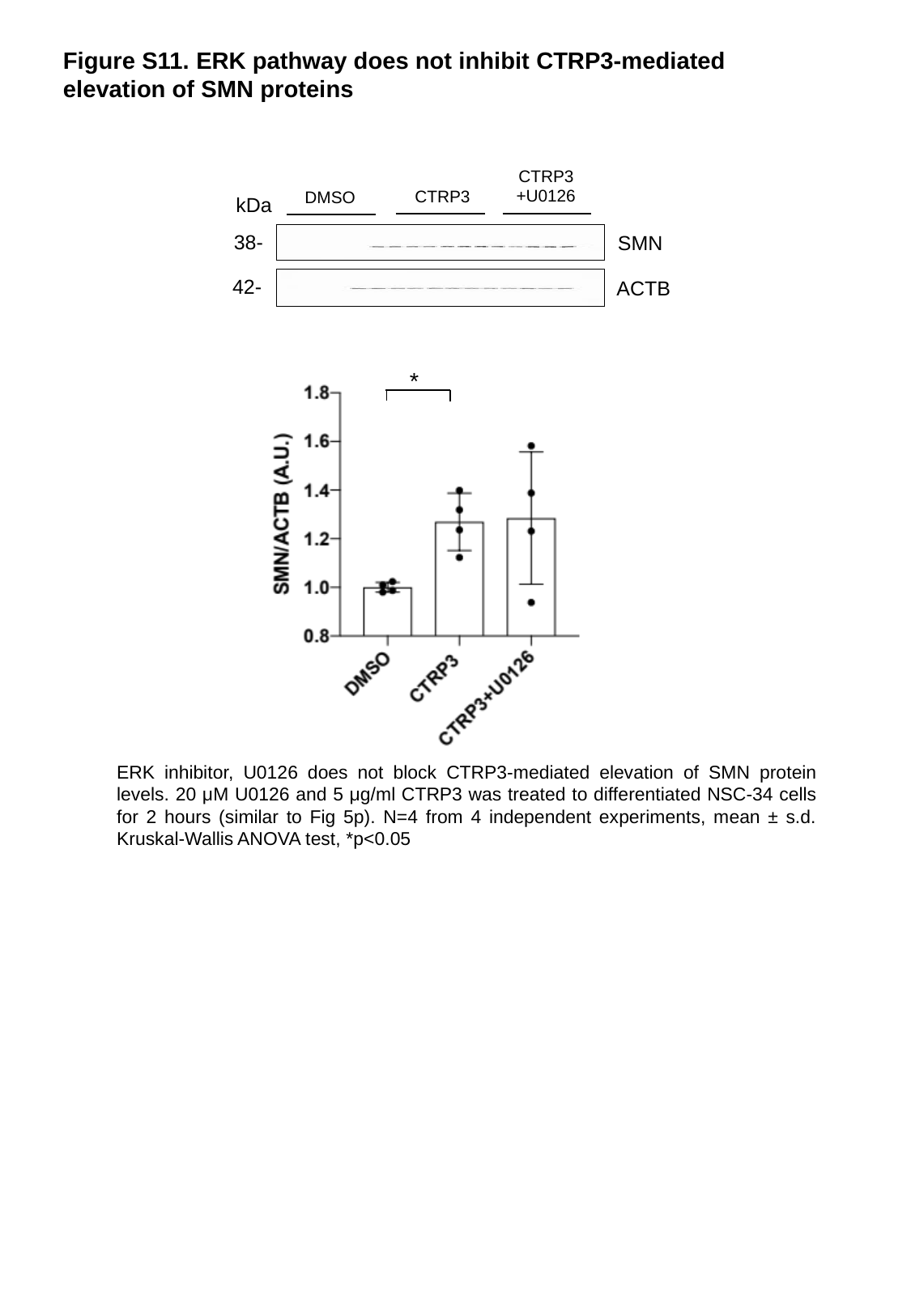

Figure S11. ERK pathway does not inhibit CTRP3-mediated elevation of SMN proteins
CTRP3
+U0126
CTRP3
DMSO
kDa
38-
SMN
42-
ACTB
*
ERK inhibitor, U0126 does not block CTRP3-mediated elevation of SMN protein levels. 20 μM U0126 and 5 μg/ml CTRP3 was treated to differentiated NSC-34 cells for 2 hours (similar to Fig 5p). N=4 from 4 independent experiments, mean ± s.d. Kruskal-Wallis ANOVA test, *p<0.05

## Slide 12
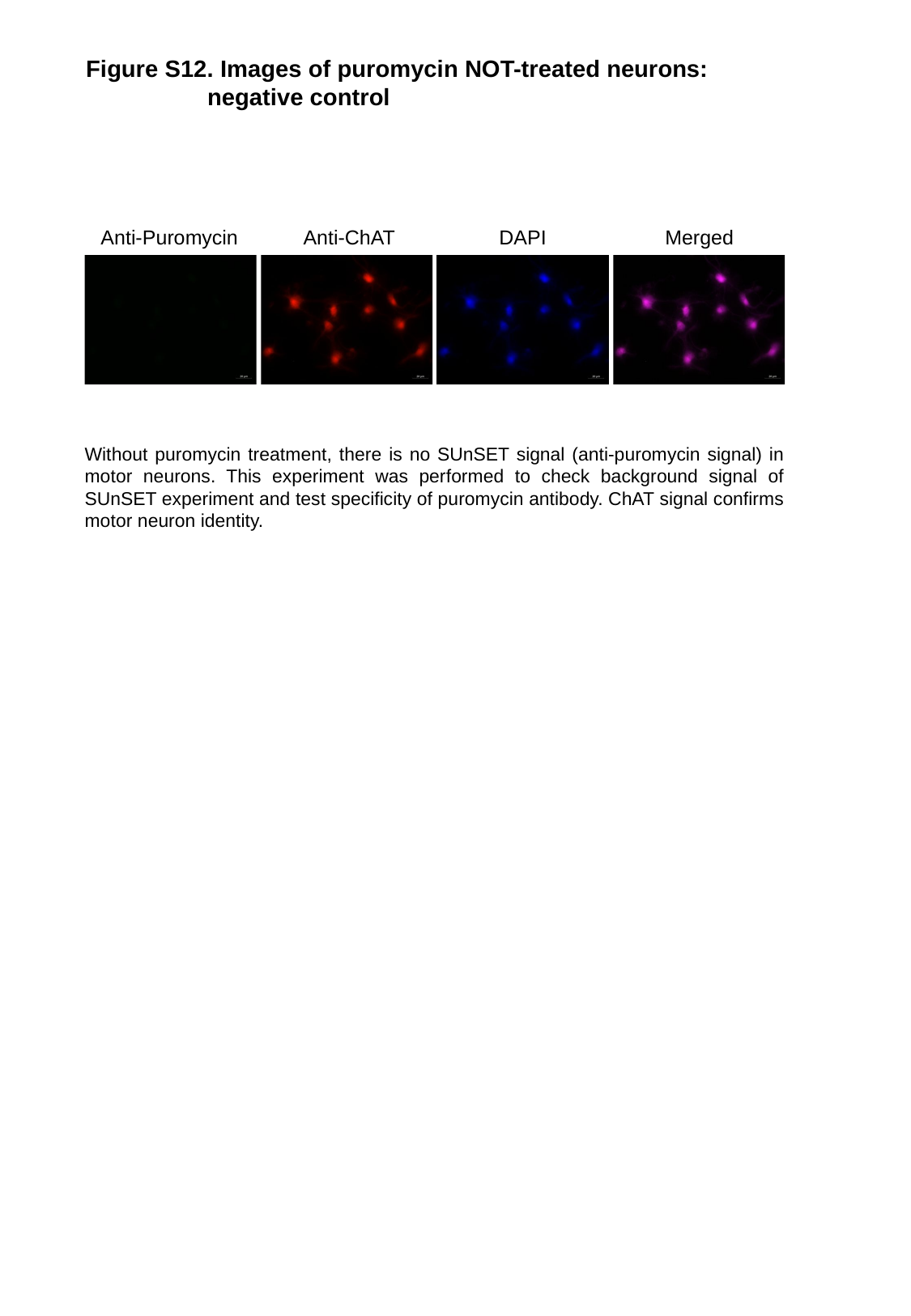

Figure S12. Images of puromycin NOT-treated neurons:
	negative control
Merged
DAPI
Anti-ChAT
Anti-Puromycin
Without puromycin treatment, there is no SUnSET signal (anti-puromycin signal) in motor neurons. This experiment was performed to check background signal of SUnSET experiment and test specificity of puromycin antibody. ChAT signal confirms motor neuron identity.

## Slide 13
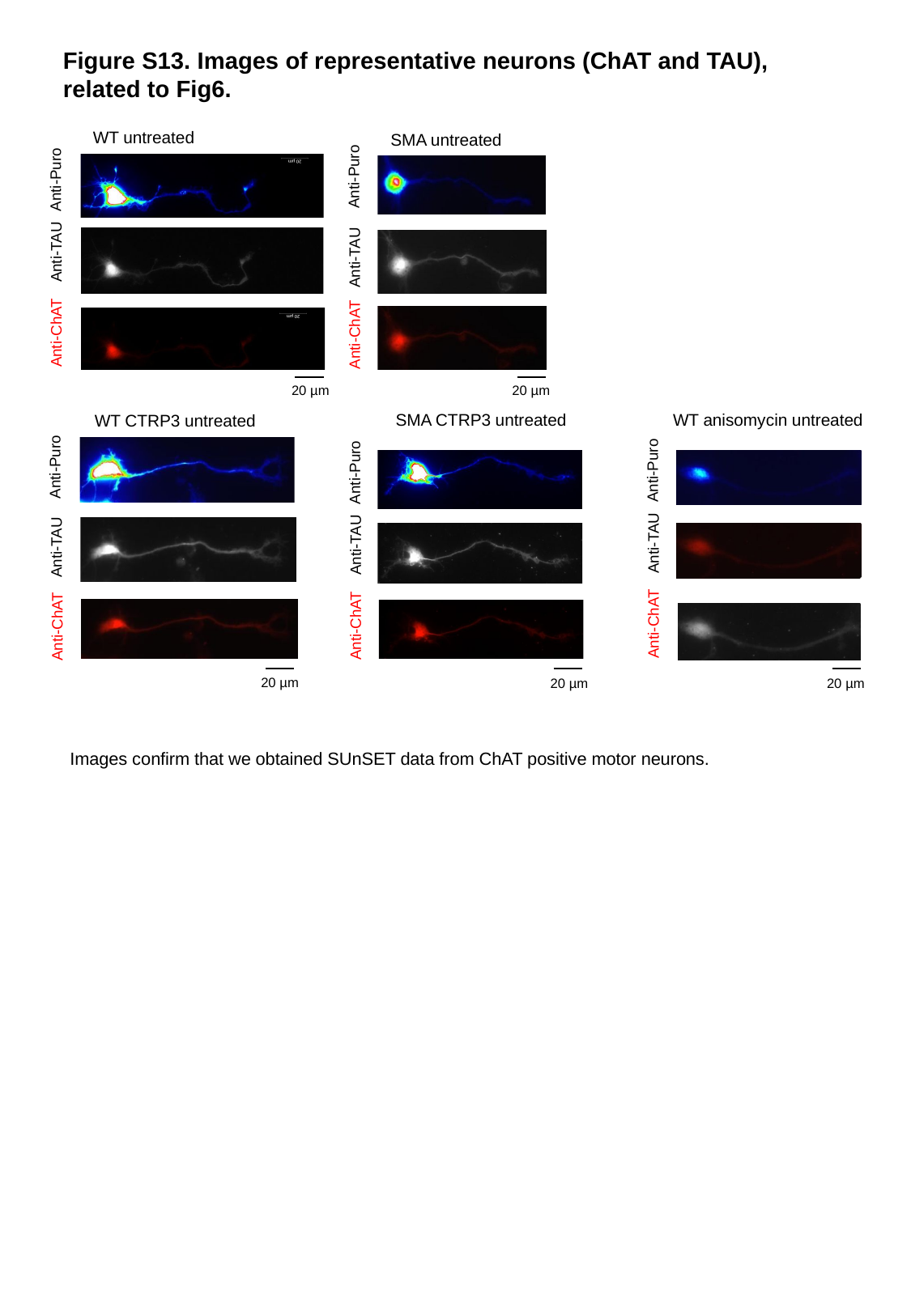

Figure S13. Images of representative neurons (ChAT and TAU), related to Fig6.
WT untreated
SMA untreated
Anti-Puro
Anti-Puro
Anti-TAU
Anti-TAU
Anti-ChAT
Anti-ChAT
20 µm
20 µm
WT anisomycin untreated
SMA CTRP3 untreated
WT CTRP3 untreated
Anti-Puro
Anti-Puro
Anti-Puro
Anti-TAU
Anti-TAU
Anti-TAU
Anti-ChAT
Anti-ChAT
Anti-ChAT
20 µm
20 µm
20 µm
Images confirm that we obtained SUnSET data from ChAT positive motor neurons.
